# The *U2AF2* /circRNA ARF1/miR-342–3p/*ISL2* feedback loop regulates angiogenesis in glioma stem cells

**DOI:** 10.1186/s13046-020-01691-y

**Published:** 2020-09-07

**Authors:** Yang Jiang, Jinpeng Zhou, Junshuang Zhao, Haiying Zhang, Long Li, Hao Li, Lian Chen, Jiangfeng Hu, Wei Zheng, Zhitao Jing

**Affiliations:** 1 Department of Neurosurgery, Shanghai Tenth People’s Hospital, Tongji University School of Medicine, Shanghai, 200072 People’s Republic of China; 2 Department of Neurosurgery, The First Hospital of China Medical University, No. 155 North Nanjing Street, Shenyang, 110001 China; 3 International Education College, Liaoning University of Traditional Chinese Medicine, No. 79 Chongshan East Road, Shenyang, 110042 China; 4 Department of Gastroenterology, Shanghai General Hospital, Shanghai Jiaotong University School of Medicine, No. 100 Haining Road, Shanghai, 20080 China; 5 Department of Histology and Embryology, College of Basic Medical Science, China Medical University, No. 77 Puhe Road, Shenyang, 110122 China

**Keywords:** Glioma stem cells, Angiogenesis, *ISL2*, circRNA ARF1, *U2AF2*, miR-342–3p

## Abstract

**Background:**

Glioma is the most common and lethal primary brain tumor in adults, and angiogenesis is one of the key factors contributing to its proliferation, aggressiveness, and malignant transformation. However, the discovery of novel oncogenes and the study of its molecular regulating mechanism based on circular RNAs (circRNAs) may provide a promising treatment target in glioma.

**Methods:**

Bioinformatics analysis, qPCR, western blotting, and immunohistochemistry were used to detect the expression levels of *ISL2*, miR-342–3p, circRNA ARF1 (cARF1), *U2AF2*, and *VEGFA*. Patient-derived glioma stem cells (GSCs) were established for the molecular experiments. Lentiviral-based infection was used to regulate the expression of these molecules in GSCs. The MTS, EDU, Transwell, and tube formation assays were used to detect the proliferation, invasion, and angiogenesis of human brain microvessel endothelial cells (hBMECs). RNA-binding protein immunoprecipitation, RNA pull-down, dual-luciferase reporter, and chromatin immunoprecipitation assays were used to detect the direct regulation mechanisms among these molecules.

**Results:**

We first identified a novel transcription factor related to neural development. *ISL2* was overexpressed in glioma and correlated with poor patient survival. *ISL2* transcriptionally regulated *VEGFA* expression in GSCs and promoted the proliferation, invasion, and angiogenesis of hBMECs via *VEGFA*-mediated *ERK* signaling. Regarding its mechanism of action, cARF1 upregulated *ISL2* expression in GSCs via miR-342–3p sponging. Furthermore, *U2AF2* bound to and promoted the stability and expression of cARF1, while *ISL2* induced the expression of *U2AF2*, which formed a feedback loop in GSCs. We also showed that both *U2AF2* and cARF1 had an oncogenic effect, were overexpressed in glioma, and correlated with poor patient survival.

**Conclusions:**

Our study identified a novel feedback loop among *U2AF2*, cARF1, miR-342–3p, and *ISL2* in GSCs. This feedback loop promoted glioma angiogenesis, and could provide an effective biomarker for glioma diagnosis and prognostic evaluation, as well as possibly being used for targeted therapy.

## Background

Glioma is the most common and lethal primary brain tumor in adults. Many treatment approaches, including surgery and radiochemotherapy are not ideal, and the average survival time of patients is less than 15 months [1, 2]. Glioma is comprised of heterogeneous cell populations, including a subpopulation of glioma stem cells (GSCs) showing tumor initiation, self-renewal, and multi-lineage differentiation abilities [3]. GSCs have been shown to be responsible for glioma proliferation, therapeutic resistance, and recurrence [4]. There is therefore a great need to identify the molecular mechanisms responsible for GSCs proliferation and progression, as well as to identify novel molecular targets for treatment of glioma.

*ISL2* is a LIM/homeodomain-type transcription factor of the Islet-1 family, and is mainly expressed in the primary sensory and motor neurons [5]. It has been reported that *ISL2* is essential for acquisition of motor neuron identity, and it contributes to the restriction of motor neurons within the neural tube via slit and semaphorin signaling [6, 7], while *ISL2* inhibition impairs peripheral axonal outgrowth in embryonic zebrafish [5]. In addition, *ISL2* participates in the formation of topographic maps in the visual system [8, 9]. *ISL1* is a member of the Islet-1 family and shares 72% protein sequence identity with *ISL2* [6]. *ISL1* participates in the development and functional regulation of sympathetic neurons, motor neurons, and retinal ganglion cells [10–12]. *ISL1* also acts as an oncogene in breast cancer, gastric cancer, and neuroendocrine carcinoma [13–15]. However, there has been no study of the possible effects of *ISL2* on cancers, including glioma. As a transcription factor involved in development of the nervous system, it is doubtful whether *ISL2* affects the development and progress of glioma.

Circular RNAs (circRNAs) have emerged as a new class of noncoding RNAs that form single-stranded closed loop structures by forming covalent bonds without the 5′ caps and 3′ poly(A) tails [16]. The circular structure of circRNAs facilitates their stable existence in different tissues, and their ability to play vital roles in multiple biological functions [17]. Moreover, studies have shown that circRNAs are dysregulated in cancers and can either promote or inhibit the proliferation, metastasis, apoptosis, and angiogenesis of cancers [18]. Mechanistically, circRNAs can either mediate transcription, interact with RNA-binding proteins, or function as competitive endogenous RNAs (ceRNAs) to regulate the expression of genes involved in tumorigenesis and progression [19–21]. Although several circRNAs have been reported in glioma, few GSCs-related circRNAs and their functions and molecular mechanisms have been clearly elucidated.

In the present study, we first identified *ISL2* as a novel oncogene in glioma, which was overexpressed and mainly involved in glioma angiogenesis via *VEGFA*-mediated ERK signaling, according to both bioinformatics analyses and molecular experiments. Moreover, we found a novel and overexpressed circRNA, cARF1 (circBase ID: hsa_circ_0016767), in GSCs, which regulated *ISL2* via sponging miR-342–3p. The cARF1 was back-spliced from the *ARF1* gene located at chr1: 228082660–228,099,212, and finally formed a sense-overlapping circular transcript of 1597 nucleotides with three exons from the *ARF1* mRNA transcript 1. MiR-342–3p is reported to exert tumor inhibiting effects in several cancers [22]. Finally, as a transcription factor, *ISL2* directly transcribed the expression of *U2AF2*, which is a type of RNA binding protein (RBP), contains a sequence-specific RNA-binding region for splicing, and promotes the stability of cARF1. Our study therefore identified a *U2AF2*/cARF1/miR-342–3p/*ISL2* feedback loop in GSCs, which promoted glioma angiogenesis, and which may provide novel targets for glioma therapy.

## Methods

### Patient samples and ethical approval

Seventy clinical samples from glioma patients were collected from January 2007 to December 2012 at the First Affiliated Hospital of China Medical University. There were 20 samples of grade II, 25 samples of grade III, and 25 samples of grade IV glioma. During the same period, 10 more acute brain injury patient samples were collected as a control group. Clinical information for these samples is outlined in Table S1. This study was approved by the Ethics Committee of the First Affiliated Hospital of China Medical University, and written informed consent was obtained from each patient.

### Cell culture and GSCs isolation

Human brain microvessel endothelial cells (hBMECs) were purchased from ScienCell Research Laboratories (San Diego, CA, USA). The hBMECs were maintained in endothelial cell medium (ECM; ScienCell Research Laboratories). Six patient-derived primary glioma stem cells from WHO grade II to IV (grade II: GSC205 and GSC207; grade III: GSC306 and GSC307; grade IV: GSC406 and GSC408) were isolated, and neurosphere cultures were obtained as previously described [23]. The detailed clinicopathological information is presented in Table S2. Briefly, freshly resected glioma samples were dissociated into single cells and grown in serum-free DMEM/F12 with 2% B27, 20 ng/mL rh-bFGF, and rh-EGF (Gibco, Gaithersburg, MD, USA). The stem cell markers of GSCs were detected by immunofluorescence using anti-CD133 (Abcam Technology, Cambridge, UK) and nestin antibodies (Abcam). The immunofluorescence staining of glial fibrillary acidic protein (GFAP; Abcam) and β-III tubulin (Abcam) was used to evaluate the multi-lineage differentiation capacity of GSCs.

### Preparation of the glioma conditioned medium (GCM)

The preparation of GCM has been previously described [24]. Briefly, we used serum-free DMEM/F12 to wash GSCs three times, followed by culturing the GSCs for 24 h. The medium was then collected and centrifuged at 3000×*g* for 15 min at 4 °C to remove GSCs and debris. The GCM was prepared and used immediately for the treatment of hBMECs, followed by subsequent experiments, or stored at − 80 °C for no more than 1 week.

### Lentiviral vector construction and transfection

The lentivirus-based vectors for *ISL2* overexpression, *U2AF2* overexpression, cARF1 overexpression, RNAi-mediated knockdown of *ISL2*, *U2AF2* and cARF1, and their negative controls were all constructed by Gene-Chem (GV358, Shanghai, China). The detailed sequence of the lentivirus-based vectors can be obtained on the GeneChem website (http://www.genechem.com.cn/index/supports/zaiti_info.html?id=50). The miR-342–3p mimic, inhibitor, and their negative controls were obtained from Thermo Fisher Scientific (Assay ID: MH12328 and MC12328; Thermo Fisher Scientific, Waltham, MA, USA). The sequences of all siRNAs are listed in Table S3. The lentivirus transfection and efficacy measurements were performed as previously described [23].

### qRT-PCR (real-time quantitative reverse transcription PCR)

Real-time PCR was performed as previously described [23]. The Mini-BEST Universal RNA Extraction kit (TaKaRa, Kyoto, Japan) was used to extract the total RNA of GSCs. For circRNA and mRNA, the RNA was reverse transcribed into cDNA using a Prime Script RT Master Mix reagent kit (TaKaRa). The qPCR assays were detected using the SYBR Green Master Mix (TaKaRa) with PCR LightCycler480 (Roche Diagnostics, Basel, Switzerland). Furthermore, RNase R (Epicentre Technologies, Madison, WI, USA) was used to confirm the existence of cARF1 and eliminate the effect of linear ARF1 RNA. The β-actin was used as an endogenous control. For miRNA, cDNA was synthesized using the PrimeScript™ RT reagent kit (TaKaRa, Shiga, Japan). The expression levels of miR-342–3p were detected using the TaqMan Universal Master Mix II (Assay ID: 002260; Applied Biosystems, Foster City, CA, USA). The U6 housekeeping gene was used as an endogenous control (Assay ID: 001973, Applied Biosystems). Primers used in this study are listed in Table S4.

### Western blotting

Western blotting was performed as previously described [23]. Briefly, the total proteins of GSCs or tissues were isolated using a total cell protein extraction kit (KeyGen Biotechnology, Nanjing, China). Protein lysates were prepared, subjected to SDS-PAGE, transferred onto polyvinylidene difluoride membranes and blocked with 2% bovine serum albumin (KeyGen Biotechnology). The primary antibodies against ISL2 (1:1000; Abcam), VEGFA (1:1000; Abcam), VEGFR2 (1:1000; Abcam), p-VEGFR2 (1:1000; Abcam), MEK1/2 (1:1000; Abcam), p-MEK1/2 (1:1000; Abcam), ERK1/2 (1:500; Abcam), p-ERK1/2 (1:500; Abcam), and β-actin (1:2000; Proteintech, Rosemont, IL, USA) were incubated at 4 °C overnight. After secondary antibody (Proteintech) incubation, the bands were detected using a chemiluminescence ECL kit (Beyotime Biotechnology, Beijing, China) and quantified by ImageJ software (National Institutes of Health, Bethesda, MD, USA).

### Immunohistochemistry (IHC)

IHC was performed and the results were semi-quantified as previously described [23]. Briefly, the paraffin-embedded tissue sections were labeled with primary antibody against ISL2 (1:100; Abcam), U2AF2 (1:100; Abcam), VEGFA (1:100; Abcam), and CD31 (1:100; Abcam). The sections were then treated with an immunohistochemical labeling kit (MaxVision Biotechnology, Fuzhou, China) and photographed with a light microscope (Olympus, Tokyo, Japan). The German immunohistochemical score was used to evaluate the staining intensity and expression levels [25].

### Immunofluorescence

Immunofluorescence staining was performed as previously described [24]. Briefly, the GSCs were fixed with 4% paraformaldehyde, permeabilized with 0.5% Triton X-100, blocked with 5% bovine serum albumin, and detected with primary antibodies against CD133, nestin, GFAP, and β-IIItubulin (1:100; Abcam) at 4 °C overnight. The samples were stained by fluorescein isothiocyanate- or rhodamine-conjugated secondary antibodies. Finally, GSCs were counterstained using 4′,6-diamidino-2-phenylindole (Sigma-Aldrich, St. Louis, MO, USA) and were visualized using a laser scanning confocal microscope (Olympus).

### Cell viability assay

The hBMECs were plated in 96-well plates at a density of 1000 cells/well and incubated in GCM for 0, 24, 48, 72, 96, and 120 h. Cell viability was determined using the CellTiter 96® Aqueous Non-Radioactive Cell Proliferation Assay Kit (Promega, Madison, WI, USA) according to the manufacturer’s instructions.

### EDU assay

The EDU assay was conducted to examine the proliferation of cells using an EDU assay kit (Beyotime, Biotechnology) according to the manufacturer’s protocol. Briefly, the hBMECs were treated with GCM and seeded into 24-well plates at 1 × 10^5^ cells/well for 24 h, then 10 μM EDU reagent was added to the medium and incubated for 2 h. After being fixed and permeabilized, the hBMECs were counterstained. The percentage of EDU positive cells was calculated using a laser scanning confocal microscope (Olympus).

### Transwell invasion assay

For the Transwell invasion assay, approximately 1 × 10^5^ hBMECs under different conditions were plated in the upper chamber (Corning, Corning, NY, USA) with a Matrigel filter (BD Biosciences, San Jose, CA, USA) and ECM medium with 10% fetal bovine serum was added to the lower chamber. After incubation for 24 h, the invaded cells were fixed with 4% paraformaldehyde and stained with Crystal Violet (Beyotime, Biotechnology). The stained cells were photographed and counted using a light microscope (Olympus).

### Tube formation assay

The tube formation assay was performed as previously described [24]. Briefly, pre-chilled 96-well plates were coated with 70 μL Matrigel filter reagent (BD Biosciences) per well at 37 °C for 30 min. The hBMECs under different conditions were seeded on the surface of the Matrigel at 2 × 10^4^ cells/well at 37 °C for 4 h. A microscope (Olympus) was used to visualize the images for each well, and Image J software was used to calculate the total number of branches and tubule lengths.

### Enzyme-linked immunosorbent assay (ELISA)

The ELISA was performed using a commercial kit (Cusabio, Stratech, UK) to detect the concentration of VEGFA in the supernatant of the GSCs medium, as previously described [25]. All results were normalized to the protein concentration in the control group.

### Luciferase reporter assay

Luciferase reporter assays were performed as previously described [24]. Briefly, the luciferase reporter plasmids (*VEGFA*-wt and *VEGFA*-mt, *ISL2*–3′-UTR-wt and *ISL2*–3′-UTR-mt, cARF1-wt and cARF1-mt, and *U2AF2*-wt and *U2AF2*-mt) were constructed by Gene-Chem (GV102). The detailed sequence can be obtained on the GeneChem website (http://www.genechem.com.cn/index/supports/zaiti_info.html?id=). The luciferase reporter plasmids were co-transfected into GSCs. After 48 h, the luciferase activities were detected using a Dual-Luciferase Reporter Assay System (Promega). Relative luciferase activity was calculated as the ratio of firefly luciferase activity to Renilla luciferase activity.

### Chromatin immunoprecipitation (ChIP) assays

ChIP assays were performed using the ChIP Assay Kit (Beyotime Biotechnology) according to the manufacturer’s instructions. The chromatin complexes were immunoprecipitated using anti-ISL2 antibody or normal rabbit IgG, and the purified DNA samples were analyzed by qPCR. The primers for ChIP qPCR are listed in Table S4.

### RNA immunoprecipitation (RIP) assay

The RIP assay was performed using the EZ-magna RIP RNA-binding Protein Immunoprecipitation kit (Millipore, Darmstadt, Germany) according to the manufacturer’s protocols. GSCs under different conditions were lysed in RIP buffer including magnetic beads conjugated with negative control IgG, anti-AgO2, or anti-U2AF2 antibodies (Millipore). After incubation with proteinase K, the immunoprecipitated RNAs were isolated. Finally, qRT-PCR was used to examine the precipitants.

### RNA pull-down assay

The Pierce Magnetic RNA Protein pull-down Kit (Thermo Fisher Scientific) was used to detect the interaction between cARF1 and U2AF2 according to the manufacturer’s suggestions. Briefly, biotinylated RNA probes were used to label purified RNA, and then the positive control (input), negative control (antisense RNA), and biotinylated RNA were mixed and co-incubated with GSCs proteins at room temperature. The RNA-protein complex was added with magnetic beads to prepare a probe-magnetic bead complex. After being washed and boiled, the complexes were detected by western blotting, using β-actin as a control.

### RNA stability measurement

GSCs were cultured in the medium containing 2 μg/ml actinomycin D (Act D, NobleRyder, China) to block the de novo RNA synthesis. Then total RNA was collected at indicated times and cARF1 expression was detected by qRT-PCR. The half-life of cARF1 was determined as the time required to reach 50% of the RNA levels before actinomycin D treatment.

### Xenograft experiments

Xenograft experiments were performed as previously described [24]. Under specific pathogenic conditions, 6-week-old female BALB/c nude mice (Beijing Vital River Laboratory Animal Technology, Beijing, China) were raised at the Laboratory Animal Center of China Medical University. GSCs under different conditions were injected (5 × 10^4^ cells per mouse) orthotopically into the mouse brains, 2 mm lateral and 2 mm anterior to the bregma using a stereotaxic instrument (*n* = 5, per group). The tumor volume was measured according to the following formula: V = (D × d^2^)/2, where D was the longest diameter and d was the shortest diameter of the tumor. All animal experiments were performed in accordance with the Animal Care Committee of China Medical University.

### Bioinformatics analysis

The data of mRNA expression, WHO grades, isocitrate dehydrogenase (*IDH*) status (*IDH 1/2*) of *ISL2* and *U2AF2*, the survival times, and status of glioma patients were obtained from the Chinese Glioma Genome Atlas (CGGA, http://www.cgga.org.cn) using the mRNA seq-693 dataset and The Cancer Genome Atlas (TCGA, http://cancergenome.nih.gov) in the HG-U133A platform. Gene set enrichment analysis (GSEA, http://www.broadinstitute.org/gsea/index.jsp) was used to analyze enrichment of a biological process or signal pathway with high versus low *ISL2* expressions. Four online databases, Starbase (http://starbase.sysu.edu.cn), TargetScan (www. targetscan.org), microRNA (http://www.microrna.org/microrna/home.do), and miRDB (http://mirdb.org) were used to predict possible miRNAs targeting *ISL2*. Starbase and circBase (http://www.circbase.org/) databases were used to predict potential circRNAs as sponges of miRNA. The Starbase database was also used to predict the proteins binding to circRNAs.

### Statistical analysis

Results are reported as the mean ± SD of at least three independent experiments. The chi-square test, two-tailed Student’s *t*-test, and one-way analysis of variance were used to compare the statistical significance among different groups. Pearson’s correlation analysis was used to assess the correlation between two groups. The survival difference was evaluated using a log-rank test and Kaplan-Meier analysis. SPSS statistical software for Windows, version 23.0 (IBM, Armonk, N. Y, USA) was performed for statistical analysis, and two-tailed *P* values < 0.05 were considered significant.

## Results

### *ISL2* is overexpressed in gliomas and correlates with poor patient survival

**Fig. 1:**
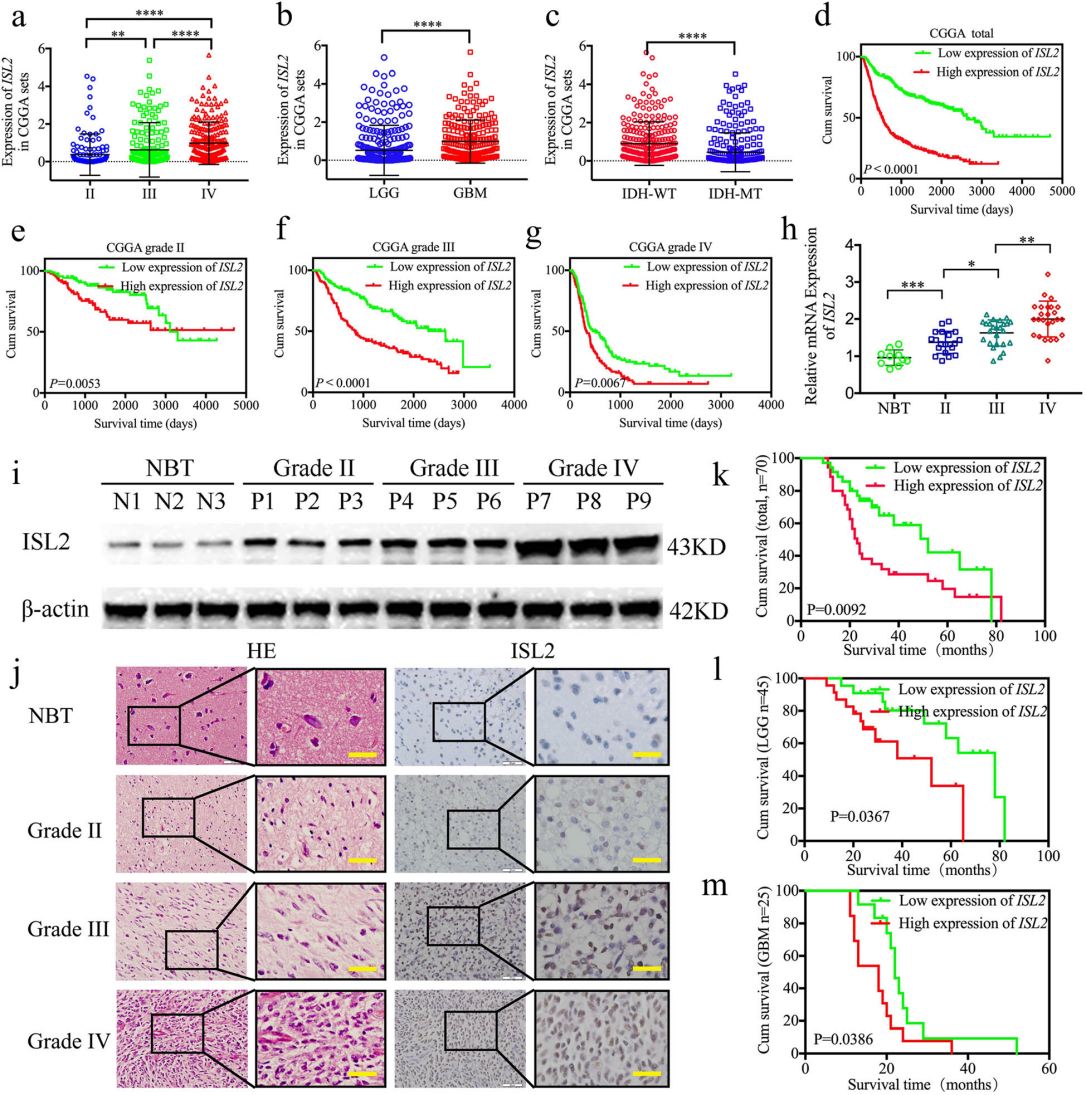
*ISL2* is overexpressed in gliomas and is correlated with poor patient survival. **a b**, **c** The mRNA expressions of *ISL2* were shown according to WHO grades (**a**), glioblastoma (GBM) and lower grade glioma (LGG) (**b**), and isocitrate de-hydrogenase (*IDH*) status (**c**) in the CGGA database. **d**, **e**, **f**, **g** The prognostic significance of *ISL2* in total grades (**d**), grade II (**e**), grade III (**f**), and grade IV (**g**) glioma tissues were detected in the CGGA database. **h**, **i**, **j** *ISL2* was expressed at higher levels in different grade glioma tissues, compared with normal brain tissue as measured by qPCR (**h**), western blots (**i**), and immunohistochemistry (**j**) (grade II, *n* = 20; grade III, *n* = 25; grade IV, *n* = 25; NBT, *n* = 10). Scale bar = 50 μm. **k**, **l**, **m** Kaplan-Meier analysis showed the prognostic significance of the 70 glioma patients, 45 LGG patients, and 25 GBM patients with high versus low *ISL2* expressions detected by qPCR. All data are expressed as the mean ± SD (three independent experiments). ^*^p < 0.05; ^**^p < 0.01; ^***^p < 0.001; ^****^p < 0.0001

To characterize the expression and functions of *ISL2* in gliomas, we first searched its expression in CGGA datasets. Compared to WHO grade II and grade III glioma, *ISL2* expression was higher in glioblastoma (GBM; WHO grade IV) (Fig. 1a, b). *ISL2* was also highly enriched in the *IDH* wildtype glioma, and was associated with decreased survival rates among different WHO grade glioma in the CGGA datasets (Fig. 1c-g). These results were also validated in TCGA datasets (Figure S1a-g). We then characterized the expressions of *ISL2* in our 70 glioma patients, 10 normal brain tissues, and patient-derived primary GSCs. Compared to the normal brain tissues, all qPCR, western blotting, and immunohistochemistry results showed that *ISL2* expression was higher in glioma tissues, and was especially increased in higher glioma WHO grades (Fig. 1h–j). Kaplan-Meier survival analyses showed that the median survival times of lower grade glioma (LGG) patients, GBM patients, or total glioma patients with higher *ISL2* expressions were all shorter than in patients with lower *ISL2* expression levels (Fig. 1k-m). Finally, both Cox univariate and multivariate analyses showed that *ISL2* expression, WHO grades, and *IDH* status were independent prognostic factors of glioma patients (Table 1).

**Table 1:** Cox Univariate and multivariate analysis of glioma patients

Factors	Categories	Univariate analysis		Multivariate analysis	
		Χ^2^	*P* value	HR	*P* value
**Gender**	Male/female	1.9916	0.1582	0.7129	0.3573
**Age**	≤50/> 50	0.1099	0.7402	0.6488	0.2530
**WHO grade**	Grade II	39.9786	**< 0.0001**	2.3579	**0.0052**
	Grade III				
	Grade IV				
**IDH status**	Mutant/wild	32.5198	**< 0.0001**	6.8621	**0.0003**
**ISL2 expression**	High/low	5.4320	**0.0198**	2.6931	**0.0193**

We successfully isolated six GSCs from glioma patients with different WHO pathological diagnoses. The original patient tumors were stained by hematoxylin and eosin (Figure S2a). The enrichment of stem cell markers, CD133 and nestin, were confirmed by immunofluorescence (Figure S2b). The multilineage differentiation capacity of GSCs and differentiation markers, GFAP and β-III tubulin, were also confirmed (Figure S2c). Both qPCR and western blotting showed that *ISL2* was expressed highest in WHO grade IV GSCs (GSC406 and GSC408), followed with WHO grade III GSCs (GSC306 and GSC307), and lowest in WHO grade II GSCs (GSC205 and GSC207) (Figure S2d, e). Moreover, we found that *ISL2* expression was higher in each GSC, when compared with the non-GSCs (Figure S2f, g). Together, these results suggested that *ISL2* was elevated in glioma and was associated with poor patient survival.

### *ISL2* transcriptionally regulates *VEGFA* expression in GSCs

**Fig. 2:**
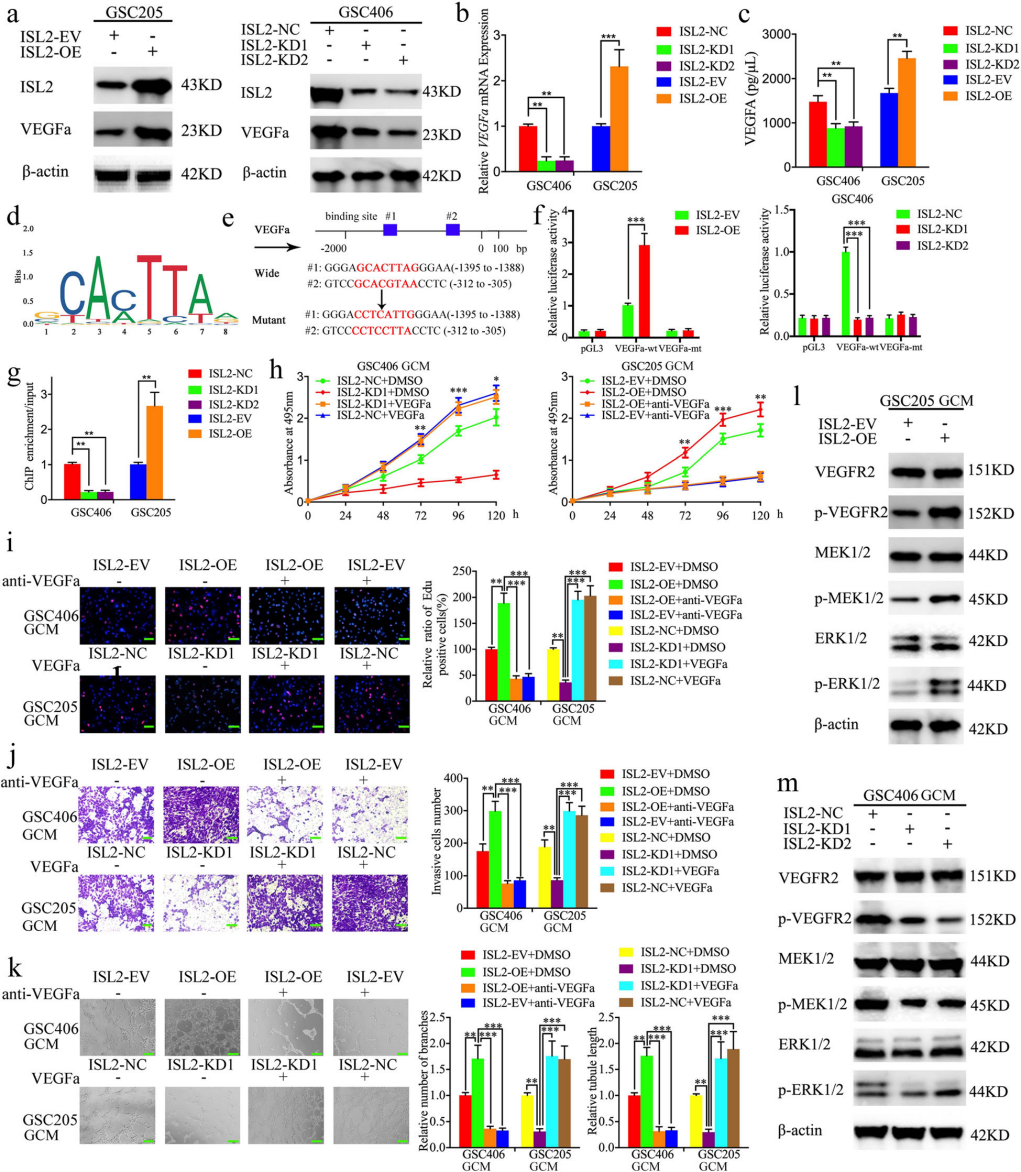
*ISL2* transcriptionally regulates *VEGFA* expression and *ISL2*-mediated glioma conditioned-medium (GCM) regulates the proliferation, invasion, and angiogenesis of hBMECs via *VEGFA* mediated ERK signaling. **a**, **b** The relative expression of *VEGFA* after *ISL2* overexpression or knockdown were detected by western blotting (**a**) and qPCR (**b**). **c** Secreted *VEGFA* levels in the GCM after *ISL2* overexpression or knockdown were measured by an ELISA. **d** Sequence motif representing the consensus *ISL2* binding motif (JASPAR database). **e** Schematic diagram of the putative *ISL2* binding site in the 3′-UTR of *VEGFA*. **f** The luciferase reporter assays showed that *ISL2* overexpression or knockdown affected the luciferase promoter activities of *VEGFA*. **g** The ChIP qPCR showed that *ISL2* bound to the promoter of *VEGFA*. **h** MTS assays showed that hBMECs cell viability with *ISL2* overexpression of GCM or *ISL2* knockdown of GCM were reversed by additional anti-*VEGFA* or recombinant *VEGFA*, respectively. **i** The EDU assay showed that the proliferation of hBMECs with *ISL2* overexpression of GCM or *ISL2* knockdown of GCM were reversed by additional anti-*VEGFA* or recombinant *VEGFA*, respectively. Scale bar = 50 μm. **j** A representative Transwell assay showed the invasion of hBMECs with *ISL2* overexpression of GCM or *ISL2* knockdown of GCM were reversed by additional anti-*VEGFA* or recombinant *VEGFA*, respectively. Scale bar = 100 μm. **k** Representative tube formation assay showed that the tubulogenesis of hBMECs with *ISL2* overexpression GCM or *ISL2* knockdown of GCM were reversed by additional anti-*VEGFA* or recombinant *VEGFA*, respectively. Scale bar = 100 μm. **l**, **m** Western blotting showed the expression of downstream targets of the ERK signaling pathway of hBMECs with *ISL2* overexpression (**l**) or knockdown (**m**) of GCM. EV: empty vector, OE: overexpression, NC: negative control, KD: knockdown. All data are expressed as the mean ± SD (three independent experiments). ^*^p < 0.05; ^**^p < 0.01; ^***^p < 0.001

To determine the possible effect of *ISL2* on glioma, we performed gene set enrichment analysis (GSEA) of *ISL2* expression based on TCGA and CGGA datasets. The results showed there was a positive association with “GO_POSITIVE_REGULATION_OF_VASCULAR_ENDOTHELIAL_ GROWTH_FACTOR_PRODUCTION” signatures in *ISL2* high expression glioma. (Fig. S1h, i). According to the expression of *ISL2* in GSCs shown in Figure S2d-e, GSC406 with the highest expression was treated for *ISL2* silencing, while GSC205 with the lowest expression was treated for *ISL2* overexpression. Lentiviral-based transfection and the effects on *ISL2* silencing or overexpression were validated in Figure S3a, b. All qPCR, western blotting, and ELISA assays showed that the expression and secretion of *VEGFA* decreased after *ISL2* silencing of GSC406, while it increased in *ISL2* overexpressing GSC205 (Fig. 2a-c). As a transcription factor, we found two binding sites for *ISL2* in the promoter of *VEGFA* when using the Jaspar database (Fig. 2d, e). A luciferase plasmid with the top 2000 nucleotides of the promoter domain of the *VEGFA* gene (pGl3-wt) and a luciferase plasmid with mutant sequences in both binding sites of the promoter domain (pGL3-mt) were generated (Fig. 2e). Luciferase reporter assays showed that *ISL2* enhanced the luciferase activity of pGL3-wt, but not that of pGL3-mt (Fig. 2f). ChIP assays also revealed that the enrichment of *VEGFA* was decreased in *ISL2* silencing GSC406 and increased in *ISL2* overexpressing GSC205 (Fig. 2g). Together, these results showed that *ISL2* transcriptionally regulated *VEGFA* expression in GSCs.

### *ISL2*-mediated GCM regulates the proliferation, invasion, and angiogenesis of hBMECs via *VEGFA*-mediated ERK signaling

We evaluated the effects of *ISL2*-regulated GCM on the proliferation of hBMECs using MTS and EDU assays. The results showed that treatment with the conditioned medium from *ISL2*-silenced GSC406 decreased cell viability and the rates of EDU-positive hBMECs, while *ISL2*-overexpressed GSC205-GCM increased the cell viability and the rates of EDU-positive hBMECs (Figure S4a–d). Transwell assays showed that treatment with *ISL2*-silenced GSC406-GCM decreased the invading cell numbers of hBMECs, whereas treatment with *ISL2*-overexpressed GSC205-GCM increased its invasive cell numbers (Figure S4e, f). Moreover, tube formation assays showed that *ISL2*-silenced GSC406-GCM treatment decreased the number of branches and tubule lengths of hBMECs, while the opposite results were obtained after treatment with *ISL2*-overexpressed GSC205-GCM (Figure S4g-i).

Taken together, the abovementioned results suggested that *ISL2* overexpression in GSCs promoted the proliferation, invasion, and angiogenesis of hBMECs. Therefore, human recombinant *VEGFA* or *VEGFA*-neutralizing antibody were combined with treatment of *ISL2*-silenced GSC406-GCM or *ISL2*-overexpressed GSC205-GCM, respectively. Both MTS and EDU assays showed that the cell viability and the rates of EDU-positive hBMECs were increased after additional human recombinant *VEGFA* treatment, when compared with *ISL2*-silenced GSC406-GCM treatment alone, while the cell viability and the rates of EDU-positive hBMECs were decreased after additional *VEGFA*-neutralizing antibody treatment, when compared with *ISL2*-overexpressed GSC205-GCM treatment alone (Fig. 2h, i). Similar results were also obtained using Transwell and tube formation assays. After additional human recombinant *VEGFA* treatment, all invading cell numbers, number of branches, and tubule lengths of hBMECs were increased when compared with *ISL2*-silenced GSC406-GCM treatment alone, whereas the opposite results were obtained after additional treatment with *VEGFA*-neutralizing antibody treatment (Fig. 2j, k). We further characterized the possible downstream *ISL2*-regulated *VEGFA* treatment on hBMECs. Western blotting showed that *ISL2*-overexpressed GSC205-GCM treatment upregulated the expression of p-VEGFR2, p-MEK1/2, and p-ERK1/2 of hBMECs (Fig. 2l), while the opposite results were obtained after treatment with *ISL2*-silenced GSC406-GCM (Fig. 2m). Together, the results showed that *ISL2* promoted angiogenesis of hBMECs in GSCs via *VEGFA*-mediated ERK signaling.

### MiR-342–3p negatively regulates *ISL2* expression through binding its 3′-UTR

**Fig. 3:**
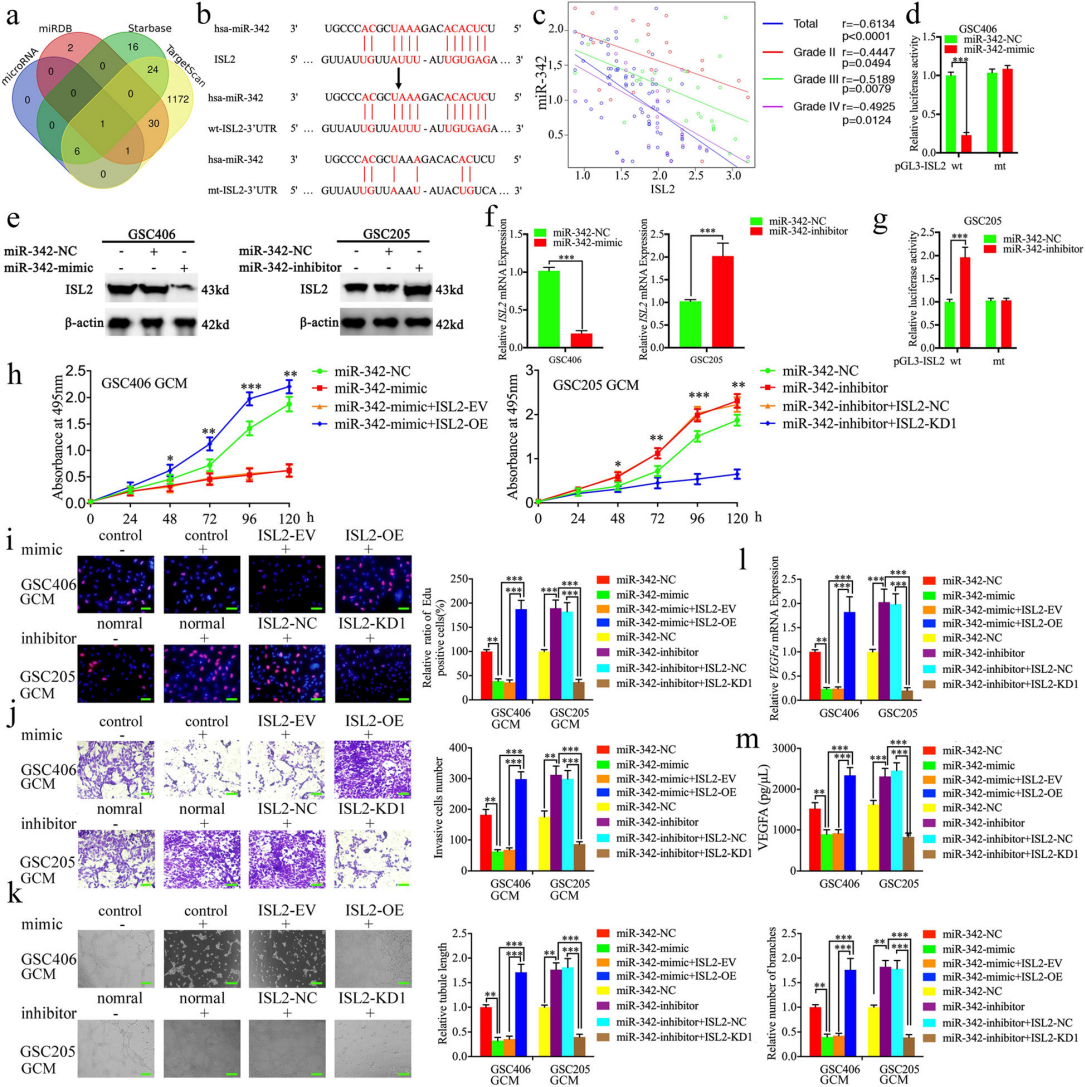
The miR-342–3p negatively regulated *ISL2* and miR-342–3p-mediated GCM suppressed the proliferation, invasion, and angiogenesis of hBMECs. **a** Identification of a miRNA that potentially regulated *ISL2* expression based on microRNA, miRDB, Starbase, and TargetScan databases. **b** Schematic diagram of the putative miR-342–3p binding site in the 3′-UTR of *ISL2*. **c** The qPCR showed the mRNA expression correlation between *ISL2* and miR-342–3p in 70 cases of glioma patients. **d**, **g** The luciferase reporter assays showed that miR-342–3p mimic (**d**) or inhibitor (**g**) altered the luciferase promoter activities of *ISL2*. **e**, **f** Western blotting (**e**) and qPCR (**f**) showed the expression of *ISL2* in glioma stem-like cells (GSCs) after miR-342–3p mimic or inhibitor treatment. **h** MTS assays showed that the miR-342–3p mimic or inhibitor transfected GCM affected hBMEC viability and was reversed by *ISL2* overexpression or knockdown, respectively. **i** The EDU assay showed that the miR-342–3p mimic or inhibitor transfected GCM affected the proliferation of hBMECs and was reversed by *ISL2* overexpression or knockdown, respectively. Scale bar = 50 μm. **g** A representative Transwell assay showed that the miR-342–3p mimic or inhibitor-transfected GCM affected the invasion of hBMECs and was reversed by *ISL2* overexpression or knockdown, respectively. Scale bar = 100 μm. **k** Representative tube formation assay showed that the miR-342–3p mimic or inhibitor-transfected GCM affected the tubulogenesis of hBMECs and was reversed by *ISL2* overexpression or knockdown, respectively. Scale bar = 100 μm. **l**, **m** The qPCR (**l**) and ELISA assay (**m**) indicated that the miR-342–3p mimic or inhibitor regulated mRNA expression and secretion of *VEGFA* in GSCs and was reversed by *ISL2* overexpression or knockdown, respectively. EV: empty vector, OE: overexpression, NC: negative control, KD: knockdown. All data are expressed as the mean ± SD (three independent experiments). ^*^p < 0.05; ^**^p < 0.01; ^***^p < 0.001

To explore which miRNA negatively regulated the expression of *ISL2*, we searched four datasets including microRNA, miRDB, TargetScan, and Starbase to identify possible miRNAs. The results showed that miR-342–3p was the only intersection among these four datasets that bound to the 3′-UTR of *ISL2* (Fig. 3a, b, Table S5). The binding site of miR-342–3p on the *ISL2* 3’UTR was predicted via Starbase, which had the highest “AgoExpNum”, “CleaveExpNum” and “Pan-Cancer” scores. We therefore designed luciferase reporter assays and found that miR-342–3p mimic treatment decreased the luciferase activity of the luciferase reporter plasmid with the wildtype *ISL2* mRNA 3′-UTR in GSC406 (Fig. 3d), while the luciferase activity of wildtype *ISL2* mRNA 3′-UTR was increased after miR-342–3p inhibitor treatment in GSC205 (Fig. 3g). We then detected the expression of miR-342–3p in our clinical glioma specimens and found its expression was negatively correlated with *ISL2* expression in each WHO grade of glioma (Fig. 3c). Both qPCR and western blotting showed the expression of *ISL2* was significantly decreased after miR-342–3p mimic treatment in GSC406, while it was upregulated after miR-342–3p inhibitor treatment in GSC205 (Fig. 3e, f). Based on these results, miR-342–3p was a possible upstream regulatory factor, which negatively regulated *ISL2* expression by binding with the *ISL2* 3′-UTR.

### MiR-342–3p suppresses the proliferation, invasion, and angiogenesis of hBMECs by inhibiting *ISL2* expression in GSCs

We detected the possible functions of miR-342–3p expression of GSCs in hBMECs. The MTS, EDU, Transwell, and tube formation assay results showed the proliferation, invasion, and angiogenesis of hBMECs were decreased after miR-342–3p mimic-transfected GSC406-GCM treatment (Fig. 3h-k). The qPCR and ELISA assays also showed that the expression and secretion of *VEGFA* were decreased in GSC406 after transfection with the miR-342–3p mimic (Fig. 3l, m). However, the opposite results were obtained after miR-342–3p inhibitor-transfected GSC205-GCM treatment (Fig. 3h-m). To further determine whether miR-342–3p inhibited these biological functions by downregulating *ISL2* expression, rescued experiments were performed with additional transfection with *ISL2* overexpression or knockdown on the basis of the miR-342–3p mimic or inhibitor treatment, respectively. Compared with miR-342–3p mimic-transfected GSC406-GCM treatment alone, additional *ISL2* overexpression-transfected GSC406-GCM increased the cell viability and the rates of EDU-positive hBMECs, the invading cell numbers of hBMECs, and the number of branches and tubule lengths of hBMECs as measured by MTS, EDU, Transwell, and tube formation assays, respectively (Fig. 3h-k). The qPCR and ELISA also showed *VEGFA* expression in GSC406 was increased after additional transfection with *ISL2* overexpression (Fig. 3l-m). However, opposite results were also obtained after additional *ISL2*-silenced transfection when compared with the miR-342–3p inhibitor-transfected GSC205-GCM treatment alone (Fig. 3h-m). Together, these data suggested that miR-342–3p suppressed the proliferation, invasion, and angiogenesis of hBMECs by inhibiting *ISL2* and *VEGFA* expression in GSCs.

### The cARF1 acts as a sponge of miR-342–3p

**Fig. 4:**
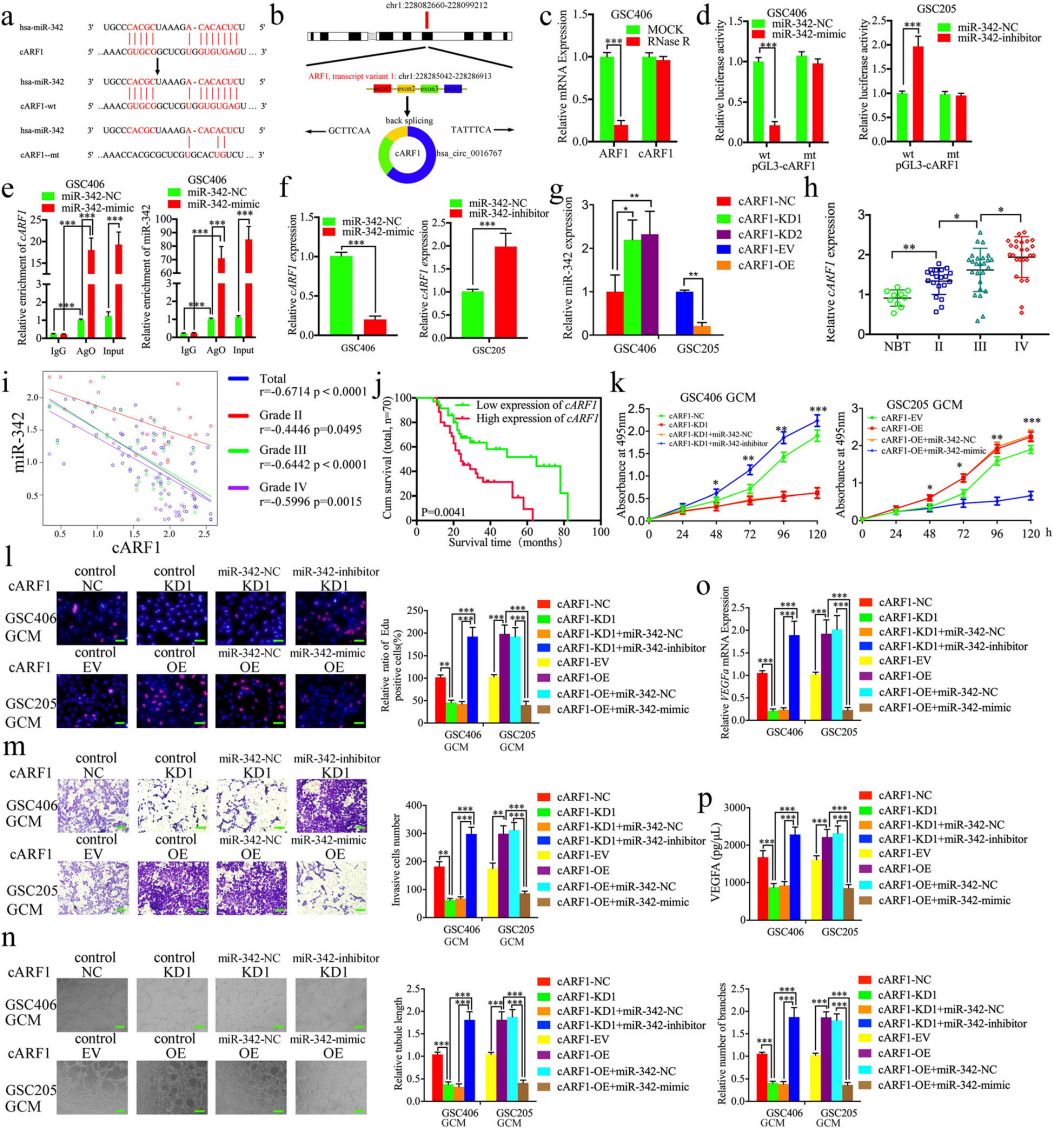
The cARF1-mediated GCM promoted proliferation, invasion, and angiogenesis of hBMECs by serving as a miRNA sponge of miR-342–3p. **a** Graphical illustration showing the predicted position of the cARF1 target on the miR-342–3p sequence. **b** A schematic representation showing that cARF1 was generated from *ARF1* gene, located at chr1. **c** The qPCR measured the relative expression of cARF1 and linear *ARF1* mRNA in GSC406 with the presence or absence of RNase R. **d** The luciferase reporter assays showed that miR-342–3p mimic or inhibitor affected the luciferase promoter activities of cARF1. **e** The anti-AgO2 RNA immunoprecipitation (RIP) assay was performed in GSC406 after the miR-342–3p mimic or negative control was transfected, followed by qPCR to detect the enrichment of cARF1 and miR-342–3p. **f** The qPCR showed the expression of cARF1 in GSCs after miR-342–3p mimic or inhibitor treatment. **g** The expression of miR-342–3p in GSCs after cARF1 overexpression or knockdown were detected by qPCR. **h** The cARF1 was expressed at higher levels in different grade glioma tissues, compared with NBT as measured by qPCR. (grade II, *n* = 20; grade III, *n* = 25; grade IV, *n* = 25; NBT *n* = 10). **i** The mRNA expression correlation between *ISL2* and cARF1 in 70 cases of glioma patients was measured by qPCR. **j** The prognostic significance of the total 70 glioma patients with high versus low cARF1 expressions as detected by qPCR. **k** MTS assays showed that cARF1 knockdown or overexpression of GCM affected hBMEC cell viability and was reversed by the miR-342–3p mimic or inhibitor treatment, respectively. **l** The EDU assay showed that cARF1 knockdown or overexpression of GCM affected the proliferation of hBMECs and was reversed by miR-342–3p inhibitor or mimic treatment, respectively. Scale bar = 50 μm. **m** A representative Transwell assay showed that cARF1 knockdown or overexpression of GCM affected the invasion of hBMECs and was reversed by the miR-342–3p inhibitor or mimic treatment, respectively. Scale bar = 100 μm. **n** A representative tube formation assay showed that cARF1 knockdown or overexpression of GCM affected the tubulogenesis of hBMECs and was reversed by the miR-342–3p inhibitor or mimic treatment, respectively. Scale bar = 100 μm. **o**, **p** The qPCR (**o**) and ELISA assay (**p**) indicated that cARF1 knockdown or overexpression of GCM regulated the mRNA expression and secretion of *VEGFA* in GSCs and was reversed by miR-342–3p inhibitor or mimic treatment, respectively. EV: empty vector, OE: overexpression, NC: negative control, KD: knockdown. All data are expressed as the mean ± SD (three independent experiments). ^*^p < 0.05; ^**^p < 0.01; ^***^p < 0.001

Increasing evidence has implied that circRNAs have many microRNA response elements (MREs), which can affect the expression and biological functions of miRNAs via competing (ceRNAs) or molecular sponges [26]. We therefore searched Starbase and found that cARF1 was the appropriate circRNA that harbors one conjectural binding site of miR-342–3p (Fig. 4a). A schematic representation showing that cARF1 was generated from the *ARF1* gene, located at chr1, is shown in Fig. 4b. RNase R is usually used to confirm the circular form of RNAs because of its ability to degrade linear RNAs with short 3′ tails, while it does not degrade circular RNAs. Figure 4c shows that the expression of *ARF1* was decreased after RNase R treatment, while there was no change in cARF1 expression. To confirm the possibility that miR-342–3p directly bound to cARF1, we constructed full-length cARF1 sequences (cARF1-wt) and cARF1 sequences with mutant binding sites (cARF1-mt) (Fig. 4a), followed by luciferase reporter assays. The results showed that the miR-342–3p mimic significantly decreased the activity of cARF1-wt vector and miR-342–3p inhibitor increased the activity of the cARF1-wt vector, while there was no change in the activity of the cARF1-mt vector group (Fig. 4d). Because miRNAs bind to MREs via the RNA-induced silencing complex (RISC), and Argo-naute 2 (AGO2) protein is the key component of RISC [17], we performed an anti-AGO2 RIP assay to determine whether miR-342–3p and cARF1 were co-enriched in the RISC. Figure 4e shows that both cARF1 and miR-342–3p were efficiently pulled down by anti-AGO2 antibody, when compared with IgG. Moreover, significant enrichment of both cARF1 and miR-342–3p were also observed after miR-342–3p mimic treatment, when compared with the miR-342–3p negative control (Fig. 4e). The qPCR also showed that the expression of cARF1 was decreased after miR-342–3p mimic treatment in GSC406, while it increased after miR-342–3p inhibitor treatment in GSC205 (Fig. 4f). In addition, lentiviral-based transfection and the effects on cARF1 knockdown or overexpression were confirmed via qPCR (Figure S3c). Overexpression or knockdown of cARF1 led to downregulation or upregulation of miR-342–3p, respectively (Fig. 4g). In summary, these results showed that cARF1 acted as a miR-342–3p sponge in GSCs.

### The cARF1 is overexpressed in glioma and correlates with poor patient survival

We examined the expression of cARF1 in our 70 glioma patients and 10 normal brain tissue samples. The qPCR results showed that cARF1 was more highly expressed in higher WHO grade glioma, while it was expressed lowest in normal brain tissues (Fig. 4h). We also performed Pearson’s correlation analyses and found that there was a negative correlation between cARF1 and miR-342–3p expressions in each WHO grade glioma and overall in all glioma samples (Fig. 4i). Kaplan-Meier survival analyses showed that glioma patients with higher cARF1 expression showed a shorter median survival time than lower expression patients (Fig. 4j).

### The cARF1 promotes the proliferation, invasion, and angiogenesis of hBMECs, while miR-342–3p reverses its function in GSCs

We detected the possible functions of cARF1 on hBMECs using the abovementioned methods. MTS, EDU, Transwell, and tube formation assays results showed that the proliferation, invasion, and angiogenesis of hBMECs were decreased after cARF1-silenced GSC406-GCM treatment (Fig. 4k-n). The qPCR and ELISA assays showed that the expression and secretion of *VEGFA* were also decreased in GSC406 after cARF1 knockdown (Fig. 4o, p), while cARF1-overexpressed GSC205-GCM treatment promoted the proliferation, invasion, and angiogenesis of hBMECs and expression and secretion of *VEGFA* in GSC205 (Fig. 4k-p). To further determine whether miR-342–3p reversed all these biological functions, rescue experiments were performed with additional treatment of the miR-342–3p mimic or inhibitor on the basis of cARF1 overexpression or knockdown, respectively. Comparing cARF1-silenced GSC406-GCM treatment alone, additional treatment of the miR-342–3p inhibitor promoted the proliferation, invasion, and angiogenesis of hBMECs, and expression and secretion of *VEGFA* of GSC406 (Fig. 4k-p). However, opposite results were also obtained after additional miR-342–3p mimic treatment, when compared with cARF1-overexpressed GSC205-GCM treatment alone (Fig. 4k-p). These results suggested that higher cARF1 expression in GSCs also promoted the proliferation, invasion, and angiogenesis of hBMECs, while miR-342–3p reversed these functions of cARF1 in GSCs.

### The cARF1 promotes gliomas angiogenesis via upregulating *ISL2* expression in GSCs

**Fig. 5:**
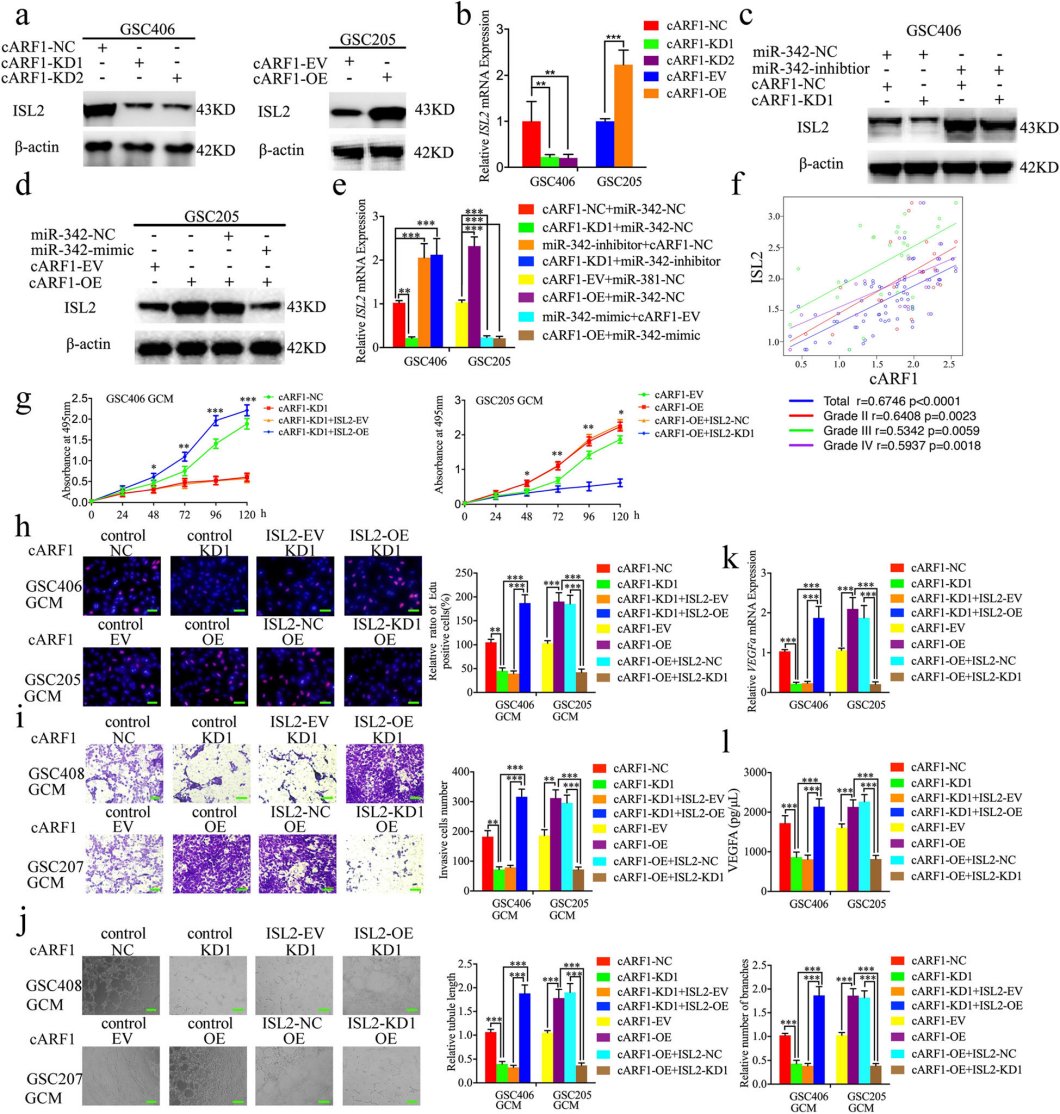
The cARF1 promoted the proliferation, invasion, and angiogenesis of hBMECs with GCM by upregulating *ISL2* expression. **a**, **b** The western blot (**a**) and qPCR (**b**) showed the expression of *ISL2* in GSCs after cARF1 overexpression (left) or knockdown (right). **c** The decreased expression of *ISL2* in GSC406 induced by cARF1 knockdown was reversed by miR-342–3p inhibitor treatment, as determined by western blotting. **d** The increased expression of *ISL2* in GSC205 induced by cARF1 overexpression was reversed by miR-342–3p mimic treatment, as determined by western blotting. **e** The effect of both cARF1 and miR-342–3p on the mRNA expression of *ISL2* in GSCs was detected by qPCR. **f** The qPCR showed the mRNA expression correlation between *ISL2* and cARF1 in 70 cases of glioma patients. **g** MTS assays showed that hBMEC cell viability with cARF1 knockdown or overexpression GCM were reversed by *ISL2* overexpression or knockdown, respectively. **h** The EDU assay showed the proliferation of hBMECs with cARF1 knockdown or overexpression of GCM were reversed by *ISL2* overexpression or knockdown, respectively. Scale bar = 50 μm. **i** A representative Transwell assay showed the invasion of hBMECs with cARF1 knockdown or overexpression of GCM were reversed by *ISL2* overexpression or knockdown, respectively. Scale bar = 100 μm. **j** A representative tube formation assay showed that the tubulogenesis of hBMECs with cARF1 knockdown or overexpression of GCM were reversed by *ISL2* overexpression or knockdown, respectively. Scale bar = 100 μm. **k**, **l** The qPCR (**k**) and ELISA assay (**l**) indicated that the mRNA expression and secretion of *VEGFA* in GSCs after cARF1 knockdown or overexpression were reversed by *ISL2* overexpression or knockdown, respectively. EV: empty vector, OE: overexpression, NC: negative control, KD: knockdown. All data are expressed as the mean ± SD (three independent experiments). ^*^p < 0.05; ^**^p < 0.01; ^***^p < 0.001

Since miR-342–3p can inhibit *ISL2* expression via binding to its 3′-UTR and cARF1 acts as a sponge of miR-342–3p, we further determined whether cARF1 regulated the expression of *ISL2* via the miR-342–3p-mediated ceRNA mechanism in GSCs. Both western blotting and qPCR showed that *ISL2* expression was overexpressed after cARF1 overexpression in GSC205 and decreased after cARF1 knockdown in GSC406 (Fig. 5a, b). We also performed rescue experiments using additional treatment of the miR-342–3p mimic or inhibitor. Western blotting and qPCR also showed the expression of *ISL2* was decreased after miR-342–3p mimic treatment in cARF1-overexpressed GSC406, while there was overexpression after miR-342–3p inhibitor treatment of cARF1-knockdown GSC406 (Fig. 5c-e). We also performed Pearson’s correlation analyses between cARF1 and *ISL2* mRNA expressions among our glioma specimens, and found that there were strong positive correlations in each WHO grade glioma and overall in all glioma samples (Fig. 5f). Rescue experiments were further performed with additional treatment of *ISL2* knockdown or overexpression on the basis of cARF1 overexpression or knockdown, respectively. Comparing the cARF1-silenced GSC406-GCM treatment alone, the MTS, EDU, Transwell, and tube formation assays results showed that the proliferation, invasion, and angiogenesis of hBMECs were increased after *ISL2* overexpression combined with cARF1-silenced GSC406-GCM treatment (Fig. 5h-j). The qPCR and ELISA assays also showed that *VEGFA* expression and secretion were increased in GSC406 after cARF1 knockdown combined with *ISL2* overexpression (Fig. 5k, l). However, the opposite results were also obtained after *ISL2* knockdown combined with cARF1 overexpression in GSC205 (Fig. 5h-l). Taken together, these results suggest that cARF1 regulates *ISL2* expression via a miR-342–3p-mediated ceRNA mechanism, and promotes gliomas angiogenesis by upregulating *ISL2* expression in GSCs.

### *U2AF2* binds to and promotes the expression of cARF1 in GSCs

**Fig. 6:**
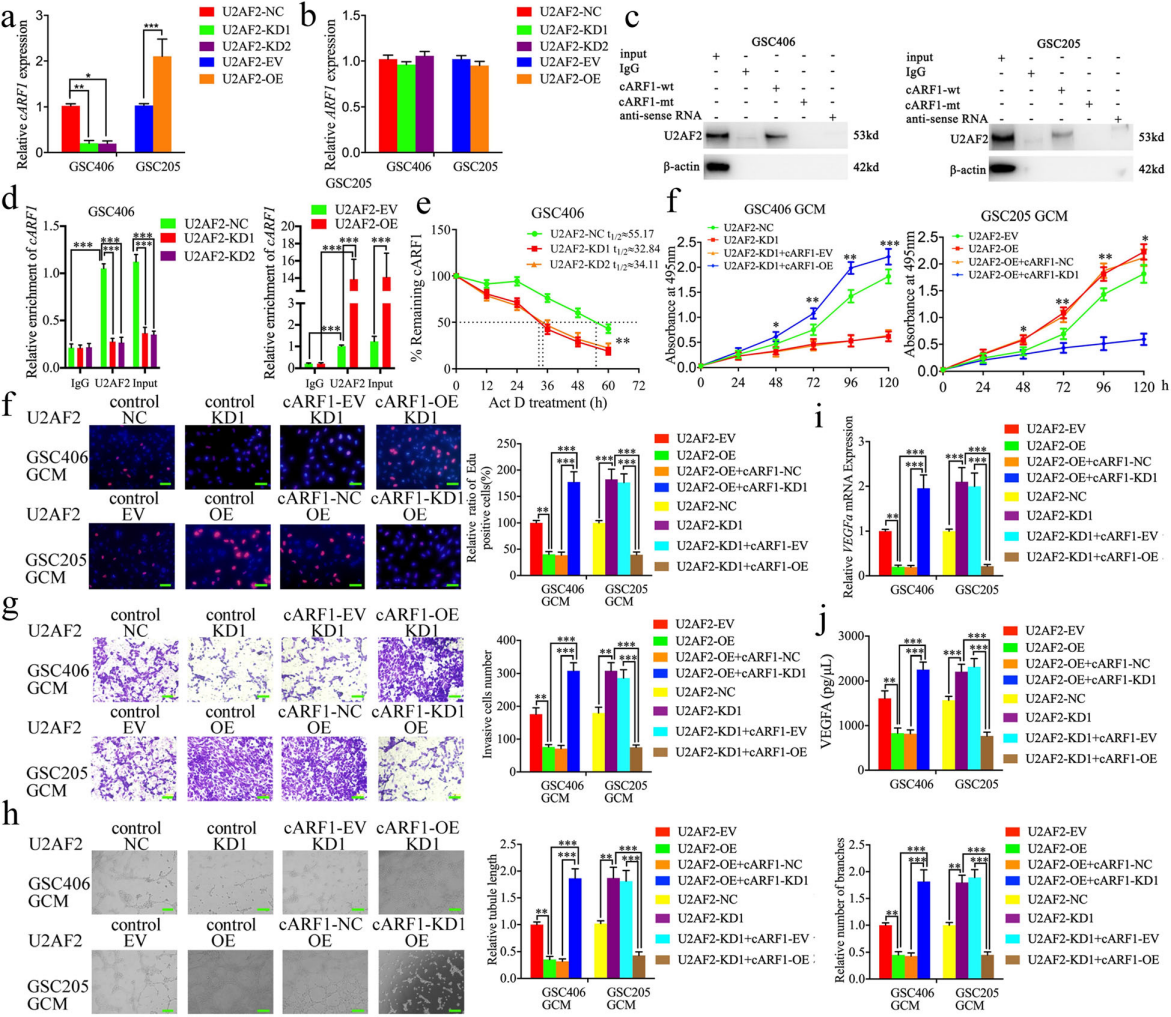
*U2AF2* bound to cARF1 and promoted glioma angiogenesis by upregulating cARF1 expression in GSCs. **a**, **b** The mRNA expression of cARF1 (**a**) and *ARF1* (**b**) after *U2AF2* knockdown or overexpression were detected by qPCR. **c** The RNA pull-down assays showed the *U2AF2* protein immunoprecipitation with cARF1 as detected by western blotting. **d** The RIP assay was performed after *U2AF2* knockdown (left) or overexpression (right), followed by qPCR to detect the enrichment of cARF1. **e** Relative expression levels of cARF1 in the *U2AF2* knockdown GSCs treated with actinomycin D at different time points were detected using qRT-PCR. **f** MTS assays showed that *U2AF2* overexpression or knockdown of GCM affected hBMEC viability and was reversed by cARF1 knockdown or overexpression, respectively. **g** The EDU assay showed that *U2AF2* overexpression or knockdown of GCM affected the proliferation of hBMECs and was reversed by cARF1 knockdown or overexpression, respectively. Scale bar = 50 μm. **h** A representative Transwell assay showed that *U2AF2* overexpression or knockdown GCM affected the invasion of hBMECs and was reversed by cARF1 knockdown or overexpression, respectively. Scale bar = 100 μm. **i** Representative tube formation assay showed that *U2AF2* overexpression or knockdown GCM affected the tubulogenesis of hBMECs and was reversed by cARF1 knockdown or overexpression, respectively. Scale bar = 100 μm. **j**, **k** The qPCR (**i**) and ELISA assay (**j**) indicated that *U2AF2* overexpression or knockdown of GCM regulated the mRNA expression and secretion of *VEGFA* in GSCs and was reversed by cARF1 knockdown or overexpression, respectively. EV: empty vector, OE: overexpression, NC: negative control, KD: knockdown. All data are expressed as the mean ± SD (three independent experiments). ^*^p < 0.05; ^**^p < 0.01; ^***^p < 0.001

Previous studies have reported that RBPs interact and regulate the expression of RNAs and contribute to the malignant behaviors of tumors [27]. We searched Starbase and found that *U2AF2* was the most probable RBP with the highest “Clip Exp Num” which could interact with cARF1. We further determined whether *U2AF2* regulated the expression of cARF1 or its linear *ARF1*. Lentiviral-based transfection and the effects on *U2AF2* knockdown or overexpression were validated as shown in Figure S3d, e using qPCR and western blotting. The qPCR results showed that *U2AF2* overexpression upregulated cARF1 expression, and that knockdown downregulated cARF1 expression, while there was no change in linear ARF1 expression (Fig. 6a, b). RNA pull-down assays were then performed to show that biotinylated cARF1-wt pulled-down *U2AF2* in GSC406 and GSC205, while cARF1-mt could not (Fig. 6c). We further performed the RIP assay to determine whether *U2AF2* bound to cARF1. The relative enrichment of cARF1 in the anti-*U2AF2* group was significantly increased when compared to that in the IgG treated group (Fig. 6d). *U2AF2* knockdown decreased the enrichment of cARF1 in GSC406, while *U2AF2* overexpression further increased the enrichment of cARF1 in GSC205 (Fig. 6d). Moreover, RNA stability measurement showed the half-life of cARF1 was obviously shortened after *U2AF2* knockdown compared with negative control group (Fig. 6e). Together, these results suggested that as a type of RBP, *U2AF2* directly promoted the stability and expression of cARF1.

### *U2AF2* is expressed at higher levels in glioma and is correlated with poor patient survival

**Fig. 7:**
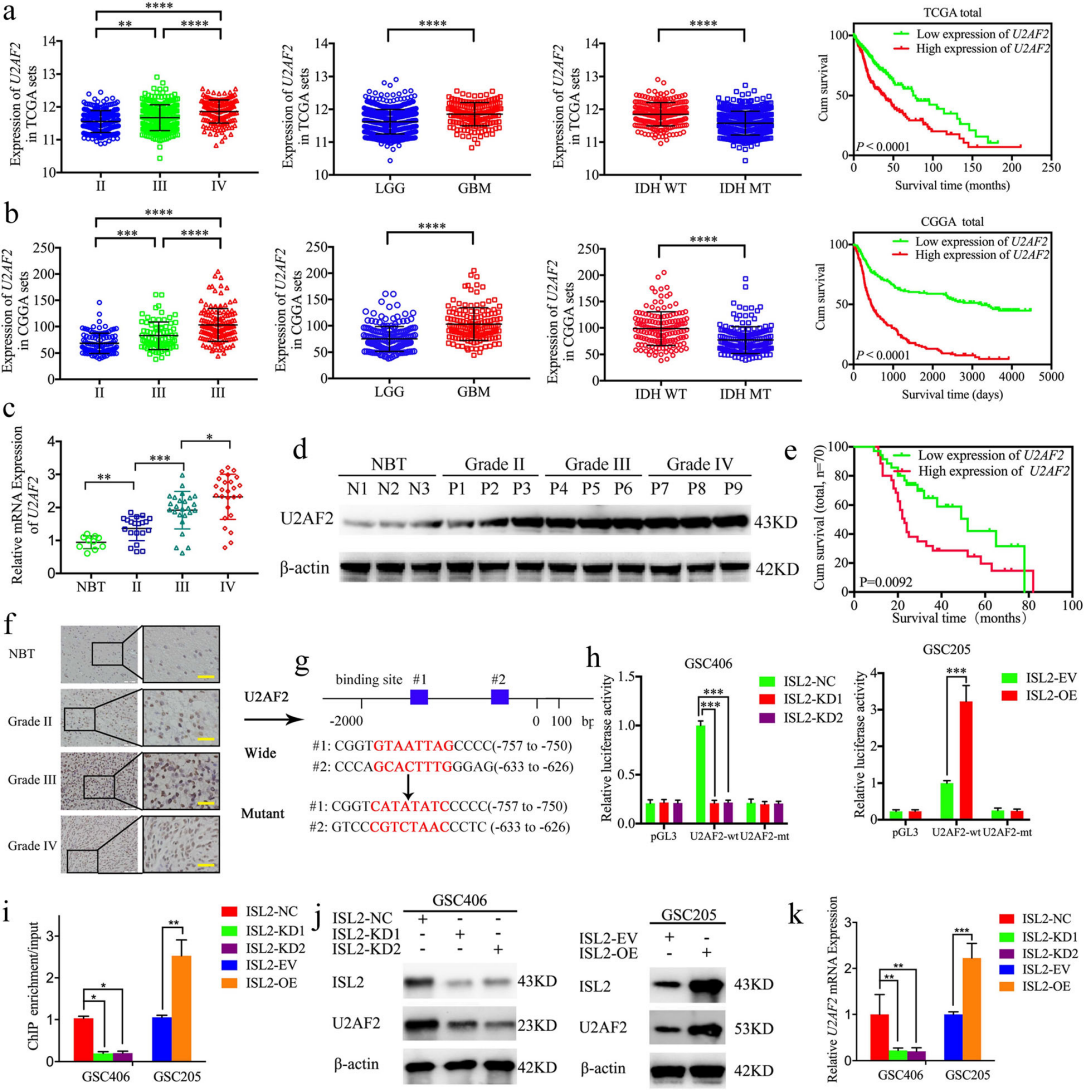
*U2AF2* was more highly expressed in gliomas, and was correlated with poor survival and transcriptionally regulated by *ISL2*. **a** The mRNA expression of *U2AF2* according to WHO grades, GBM, and LGG, IDH status, and the prognostic significance of *U2AF2* were shown in TCGA database. **b** The mRNA expression of *U2AF2* according to WHO grades, GBM and LGG, IDH status, and the prognostic significance of *U2AF2* were shown in the CGGA database. **c**, **d**, **f** *U2AF2* was expressed at higher levels in different grades of glioma tissues, compared with NBT as determined by qPCR (**c**), western blotting (**d**) and immunohistochemistry (**f**). (grade II, *n* = 20; grade III, *n* = 25; grade IV, *n* = 25; NBT *n* = 10). Scale bar = 50 μm. **e** Kaplan-Meier analysis showed the prognostic significance of 70 glioma patients with high versus low *U2AF2* expressions detected by immunohistochemistry. **g** Schematic diagram of the putative *ISL2* binding site in the 3′-UTR of *U2AF2*. **h** The luciferase reporter assays showed that *ISL2* knockdown (left) or overexpression (right) altered the luciferase promoter activities of *U2AF2*. **i** The ChIP qPCR showed that *ISL2* bound to the promoter of *U2AF2*. **j**, **k** The western blot (**j**) and qPCR (**k**) showed that the expression of *U2AF2* in GSCs after *ISL2* knockdown (left) or overexpression (right). EV: empty vector, OE: overexpression, NC: negative control, KD: knockdown. All data are shown as the mean ± SD (three independent experiments). ^*^p < 0.05; ^**^p < 0.01; ^***^p < 0.001

To characterize the expression and functions of *U2AF2* in glioma, we searched its expression in both TCGA and CGGA datasets. The results showed that *U2AF2* was expressed highest in WHO grade IV gliomas and GBM, and lowest in WHO grade II and LGG (Fig. 7a, b). *U2AF2* was also highly enriched in IDH wildtype gliomas, and was associated with decreased survival rates in both TCGA and CGGA datasets (Fig. 7a, b). We further examined its expression in our 70 glioma patients and 10 normal brain tissues. All qPCR, western blotting, and immunohistochemistry results confirmed higher *U2AF2* expressions in glioma tissues than in normal brain tissues, with the highest expression in WHO grade IV gliomas (Fig. 7c, d, f). Kaplan-Meier survival analyses also showed that the median survival time of higher *U2AF2* expression patients was shorter than those patients with lower *U2AF2* expression levels (Fig. 7e). Together, these results suggested that *U2AF2* was also more highly expressed in gliomas tissues, and was correlated with poor patient survival.

### *U2AF2* promotes glioma angiogenesis by upregulating cARF1 expression in GSCs

To determine whether *U2AF2* promotes glioma angiogenesis*,* we performed MTS, EDU, Transwell, and tube formation assays and found that the proliferation, invasion, and angiogenesis of hBMECs were decreased after *U2AF2*-knockdown GSC406-GCM treatment (Fig. 6e-i). The qPCR and ELISA assay results showed the expression and secretion of *VEGFA* were also decreased in GSC406 after *U2AF2* knockdown (Fig. 6j, k), while *U2AF2*-overexpressed GSC205-GCM treatment promoted the proliferation, invasion, and angiogenesis of hBMECs, and expression and secretion of *VEGFA* in GSC205 (Fig. 6e-i). Rescue experiments were also performed with additional treatment of cARF1 overexpression or knockdown on the basis of *U2AF2* knockdown or overexpression, respectively. Compared to *U2AF2*-silenced GSC406-GCM treatment alone, the results showed that the proliferation, invasion, and angiogenesis of hBMECs were increased after cARF1 overexpression when combined with *U2AF2*-silenced GSC406-GCM treatment (Fig. 6j-k). The qPCR and ELISA assays also showed that *VEGFA* expression and secretion were increased in GSC406 after *U2AF2* knockdown when combined with cARF1 overexpression (Fig. 6j, k). However, *U2AF2* overexpression combined with cARF1 knockdown in GSC205 showed the opposite results (Fig. 6f-k). Taken together, these results suggested that *U2AF2* promoted glioma angiogenesis via upregulating cARF1 expression in GSCs.

### *ISL2* transcriptionally regulates *U2AF2* expression in GSCs to form a feedback loop

Because *ISL2* is a transcription factor, we determined whether *ISL2* transcriptionally regulated *U2AF2* expression in GSCs. The Jaspar database showed that there existed two binding sites for *ISL2* in the promoter of *U2AF2* (Fig. 7g). Luciferase reporter assays were then performed to show that *ISL2* overexpression enhanced the luciferase activity of pGL3-*U2AF2*-wt, and *ISL2* knockdown decreased the luciferase activity of pGL3-*U2AF2*-wt, but not that of pGL3-*U2AF2*-mt (Fig. 7h). ChIP assays also showed that the enrichment of *U2AF2* was decreased in *ISL2*-knockdown GSC406 and increased in *ISL2*-overexpressed GSC205 (Fig. 7i). Finally, western blotting and qPCR showed that *ISL2* upregulated the expression of *U2AF2* (Fig. 7j, k). Together, these results show that *ISL2* transcriptionally regulates *U2AF2* expression in GSCs and forms a feedback loop.

### The *U2AF2*/cARF1/miR-342–3p/*ISL2* axis regulates glioma tumorigenesis and angiogenesis in vivo

**Fig. 8:**
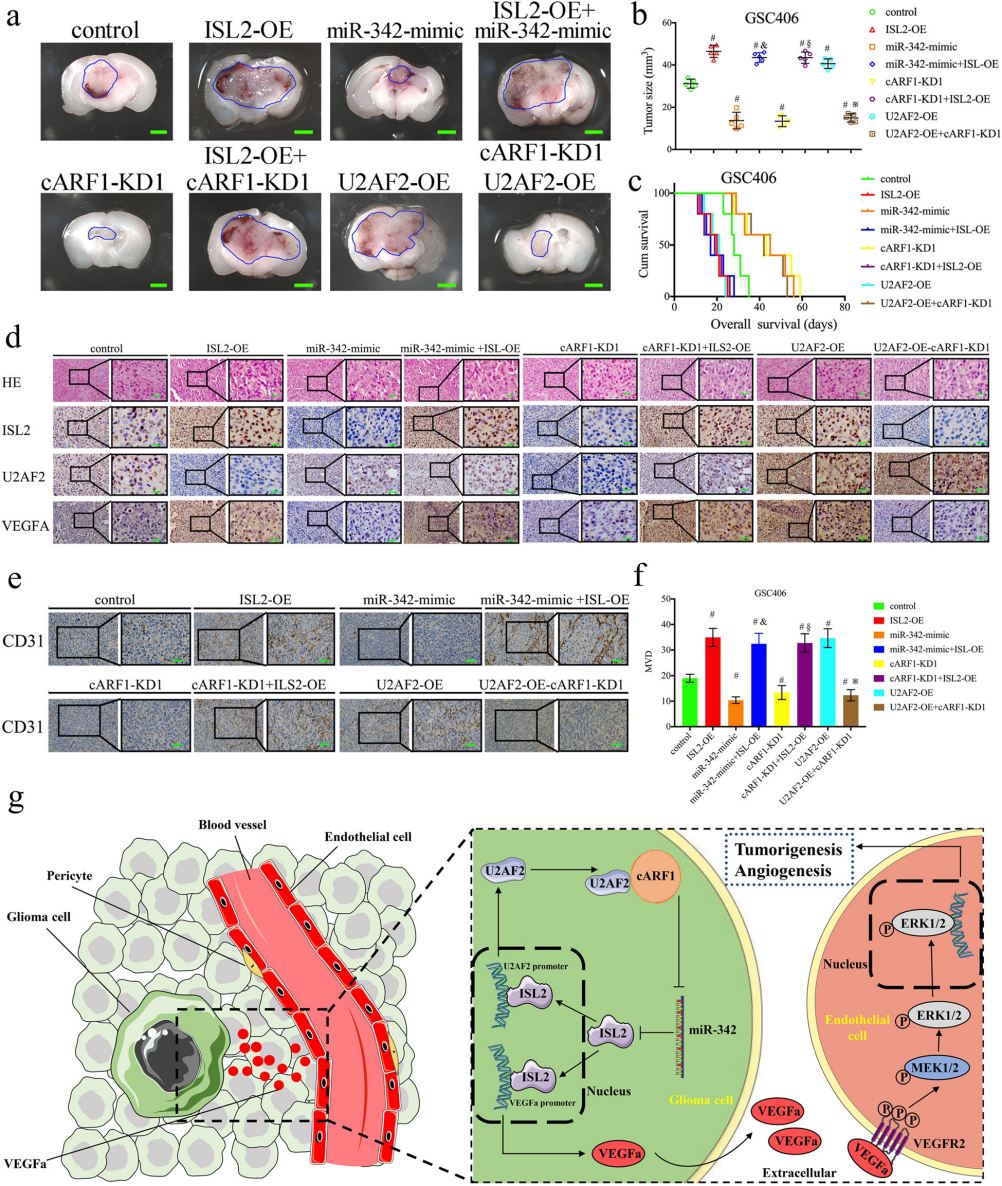
The *U2AF2*/circARF1/miR-342–3p/*ISL2* feedback loop promoted glioma tumorigenesis and angiogenesis. **a** Representative images show the size of intracranial tumors in the coronal location of eight groups (negative control, *ISL2* overexpression, miR-342–3p mimic, *ISL2* overexpression combined with miR-342–3p mimic, cARF1 knockdown, *ISL2* overexpression combined with cARF1 knockdown, *U2AF2* overexpression, and cARF1 knockdown combined with *U2AF2* overexpression in GSC406). Scale bar = 10 mm. **b** The measured tumor volumes among eight GSC406 groups are indicated. **c** Kaplan-Meier survival curves showed that *ISL2* overexpression, *U2AF2* overexpression, and *ISL2* overexpression combined with the miR-342–3p mimic in GSC406 cells shortened the survival times of nude mice, while it prolonged the survival times after the miR-342–3p mimic was transfected, cARF116 knockdown, and *U2AF2* overexpression combined with cARF116 knockdown in GSC406 cells. For each group, *n* = 5. **d**, **e** Representative immunohistochemical staining showing the changes in *ISL2*, *U2AF2*, *VEGFA*, and CD31 in the negative control, *ISL2* overexpression, miR-342–3p mimic, *ISL2* overexpression combined with miR-342–3p mimic, cARF1 knockdown, *ISL2* overexpression combined with cARF1 knockdown, *U2AF2* overexpression, and cARF1 knockdown combined with *U2AF2* overexpression orthotopic xenograft models. Scale bar = 50 μm. **f** The microvessel density (MVD) with mouse specific CD31 staining in tumor tissues were counted. **g** Schematic diagram showing that the *U2AF2*/cARF1/miR-342–3p/*ISL2* axis promoted glioma tumorigenesis and angiogenesis through *VEGFA*-mediated ERK signaling pathway. ^#^*p* < 0.05 vs. the negative control group, ^&^*p* < 0.05 vs. the miR-342–3p mimic group, ^§^*p* < 0.05 vs. the cARF1 knockdown group, ^※^*p* < 0.05 vs. the *U2AF2* overexpression group. EV: empty vector, OE: overexpression, NC: negative control, KD: knockdown

Finally, we performed orthotopic xenografts to determine the effects of the *U2AF2*/cARF1/miR-342–3p/*ISL2* axis in glioma tumorigenesis and angiogenesis in vivo. Compared to the control group, the tumor volumes were enlarged in the *ISL2* overexpression and *U2AF2* overexpression groups, and decreased in the miR-342–3p-mimic and cARF1-knockdown groups (Fig. 8a, b). Moreover, *ISL2* overexpression combined with the miR-342–3p mimic group also showed enlarged tumor volumes, while it was decreased in the *U2AF2*-overexpression combined with the cARF1-knockdown group (Fig. 8a, b). Similar results were obtained using Kaplan-Meier survival analysis, as the *ISL2*-overexpression and *U2AF2*-overexpression groups, and *ISL2*-overexpression combined with the miR-342–3p-mimic group showed shorter median survival times (MST) compared with the normal control group, while the miR-342–3p-mimic, cARF1-knockdown, and *U2AF2*-overexpression combined with cARF1-knockdown groups showed longer mean survival times (Fig. 8c). Immunohistochemistry was performed to detect the effects of the *U2AF2*/cARF1/miR-342–3p/*ISL2* axis on tumor tissues. The results showed that the *ISL2*-overexpression, *U2AF2*-overexpression, and *ISL2*-overexpression combined with the miR-342–3p-mimic or cARF1-knockdown groups showed higher expression of *VEGFA*, CD31 and increased microvessel density (MVD) (as indicated by anti-CD31 staining), while the lowest expression was found in the miR-342–3p-mimic, cARF1-knockdown, and *U2AF2*-overexpression combined with the ARF1-knockdown groups (Fig. 8d-f). Finally, we detected the mRNA expression of CD31 in our glioma specimens, and found that there were positive correlations between CD31 and cARF1, *ISL2* and *U2AF2*, while there was a negative correlation between CD31 and miR-342–3p (Figure S5). We also study the direct function of these candidate genes on the GSC properties. All, MTS, EDU and Transwell assays showed the overexpression of ILS2, cARF1 and *U2AF2* can promote the proliferation and metastasis of GSCs (Figure S6). A schematic diagram showing that the *U2AF2*/cARF1/miR-342–3p/*ISL2* feedback loop promotes glioma tumorigenesis and angiogenesis through *VEGFA*-mediated ERK signaling pathway is presented in Fig. 8g. Taken together, these results showed that the *U2AF2*/cARF1/miR-342–3p/*ISL2* axis regulated glioma tumorigenesis and angiogenesis in nude mice.

## Discussion

In this study, we first showed that *ISL2* was overexpressed in glioma and correlated with poor patient survival using bioinformatics analysis and our clinical specimens. As a transcriptional component mainly involved in the development and function of motor and sensory neurons, it is reasonable that *ISL2* may exert possible effects on the central nervous system and glioma [28]. Our study showed that *ISL2* shared a similar oncogenic role as its family member, *ISL1*, in other cancers [29, 30]. Due to its rapid and infiltrating growth properties, glioma shows active metabolism and uses an abundant blood supply in tumor tissues [27, 31]. These properties also lead to complete surgical resection and tumor recurrence [32]. Active angiogenesis is frequently observed in glioma, which can further promote its proliferation and aggressiveness [33]. Our study revealed that *ISL2* transcriptionally regulated *VEGFA* expression and promoted *VEGFA* secretion in GSCs, and that *ISL2*-mediated GCM promoted the proliferation, invasion, and angiogenesis of hBMECs via ERK signaling. We therefore conclude that the oncogenic effects of *ISL2* in glioma involved promotion of angiogenesis. Moreover, because anti-angiogenic treatment, represented by anti-VEGF therapy, such as bevacizumab, is one of the most important strategies for glioma treatment [34], *ISL2* may act as a possible therapeutic target.

Accumulating evidence has recently indicated that there are numerous circRNAs expressed in neuronal tissues, and that dysregulation of circRNAs can lead to diseases of the nervous system, including glioma [35]. The detailed regulatory molecular mechanisms of circRNAs include direct transcription and translation into functional proteins, transcriptional, and splicing regulation as well as miRNAs and RBP sponges [21, 27, 36]. For example, circ-FBXW7 encodes a novel 21 kDa protein called FBXW7-185aa in glioma, which inhibits proliferation and cell cycle acceleration [21]. Circular RNA MAPK4 (circ-MAPK4) inhibits glioma cell apoptosis via the MAPK signaling pathway by sponging miR-125a-3p in glioma [36]. Circ_002136 can bind to a RBP, *FUS*, and this regulates angiogenesis via the miR-138-5p/SOX13 axis in glioma [27]. Among these mechanisms, circRNA-mediated miRNA and RBP sponges are currently the most extensively studied. Our study therefore focused on *ISL2* regulation by circRNAs via miRNA and RBP sponges.

MiR-342–3p was the only candidate miRNA that we predicted could target the 3′-UTR of *ISL2*, based on four datasets including microRNA, miRDB, TargetScan, and Starbase. Although there has been no previous study on the regulation between miR-342–3p and *ISL2*, miR-342–3p has been reported to play an anti-tumor role in several cancers including glioma. For example, miR-342–3p expression levels have been negatively correlated with advanced WHO grades and inhibit the progression of glioma by directly targeting *PAK4* [22]. MiR-342–3p can also inhibit the malignant biological behaviors of glioblastoma cells via *Zic4* [37]. Our study further showed that miR-342–3p exerted anti-glioma effects by inhibiting GSC-GCM-mediated angiogenesis in hBMECs. Moreover, we also showed that miR-342–3p downregulated *ISL2* expression in GSCs and inhibited the angiogenesis mediated by *ISL2*.

ADP ribosylation factor 1 (*ARF1*) is a GTPase that is involved in vesicle trafficking and the Golgi apparatus [38, 39]. It was reported that *ARF1* gene promoter methylation is associated with *EGFR* gene amplification and can promote the distinct tumor infiltration in glioblastoma [38]. *ARF1* promotes cancer stem cell viability via lipid metabolism, and its ablation induces anti-tumor immune responses in mice [40]. Our study found a novel circRNA, cARF1 (hsa_circ_0016767), which was back-spliced from transcript one of *ARF1* mRNA and comprised its second, third, and fourth exons. cARF1 was overexpressed in our glioma specimens, was positively correlated with poor patient survival, and also promoted proliferation, invasion, and angiogenesis of hBMECs via *VEGFA* signaling. Moreover, as a circRNA, we also showed cARF1 had strong miRNA sponging ability toward miR-342–3p. All these results showed that cARF1 upregulated *ISL2* expression in GSCs via sponging miR-342–3p.

The RBPs are a group of more than 800 proteins, which have been identified and mainly involved in post-transcriptional regulation of RNAs, gene transcription, and translation, and participate in both physiological and pathological processes and diseases [41]. Studies of circRNA biogenesis have shown that RBP participates in circRNA splicing and expression [42]. For example, RBP binds to the introns of circRNAs linear genes near splice sites and promotes the production of circRNAs [43]. RBPs can therefore serve as an essential element underlying the functions of circRNAs, especially circRNA-mediated gene transcriptional regulation [44]. In our study, we assessed the possible effects of RBPs on the regulation of cARF1 via bioinformatics predictions, which showed that *U2AF2* was an appropriate candidate for experimental molecular validation. *U2AF2* is a spliceosome factor and a non-snRNP protein required for the binding of U2 snRNP to the pre-mRNA branch site [45]. Our study showed that *U2AF2* binds to and promotes the stability and expression of cARF1 in GSCs, while there was no effect on the expression of its ARF1 linear form.

*U2AF2* has been reported to act as an oncogene in several cancers. Bioinformatics analysis suggested that *U2AF2* was upregulated in *IDH*-mutated glioma with malignant transformation [46]. *U2AF2* expression was significantly upregulated in primary non-small cell lung cancer and was associated with metastasis, advanced tumor stages, poor survival, and recurrence [47]. In addition, *U2AF2* is significantly increased in melanoma progression and participates in brain metastasis [48]. Our study also focused on the relationship between *U2AF2* and glioma, and we showed that *U2AF2* was also a novel oncogene in glioma, because it was expressed at higher levels in glioma correlated with poor patient survival. Furthermore, *U2AF2* can also lead to the proliferation, invasion, and angiogenesis of hBMECs via upregulating cARF1 in GSCs. As a transcription factor, we showed that *ISL2* transcribed the expression of *U2AF2*, thus establishing a feedback loop among *U2AF2*, cARF1, miR-342–3p, and *ISL2* in GSCs. This feedback loop may not only promote glioma angiogenesis, but may also promote the tumorigenesis, aggressiveness, and malignant transformation, which all need to be investigated in further studies.

## Conclusions

We identified a novel transcription factor related to neural development. *ISL2* was overexpressed in glioma and correlated with poor patient survival. Using patient-derived GSCs, we found that *ISL2* transcriptionally regulated *VEGFA* expression in GSCs and promoted the proliferation, invasion, and angiogenesis of hBMECs via *VEGFA*-mediated ERK signaling. Mechanistically, cARF1 upregulated *ISL2* expression in GSCs via miR-342–3p sponging. Furthermore, *U2AF2* bound to and promoted the expression of cARF1, while *ISL2* also transcribed the expression of *U2AF2*, which formed a feedback loop and possibly participated in the malignant transformation of glioma. Moreover, we also showed both *U2AF2* and cARF1 had oncogenic effects, were overexpressed in gliomas, and correlated with poor patient survival. Our study suggested novel biomarkers for glioma diagnosis and prognosis evaluation, as well as targets for therapeutic treatment.

## Supplementary information

**Additional file 1: Supplementary Figure 1.** *ISL2* is highly expressed in glioma tissues and correlated with poor survival in TCGA database. a, b, c: The mRNA expressions of *ISL2* are shown according to WHO grades (a), GBM and LGG (b), and IDH status (c) in TCGA database. d, e, f, g: The prognostic significance of *ISL2* in total grades (d), grade II (e), grade III (f) and grade IV (g) glioma tissues were detected in TCGA database. h, i: Gene set enrichment analysis indicated that higher expression of *ISL2* was associated with the positive regulation of *VEGFA* production in both TCGA (h) and CGGA (i) databases.

**Additional file 2: Supplementary Figure 2.** Isolation and validation of patient-derived GSCs. a: Hematoxylin and eosin staining of the original patient tissues. b: Immunofluorescence staining of CD133 and nestin in patient-derived GSCs. Scale bar = 20 μm. c: Representative images showing that GSCs were differentiated and adherent (above). Scale bar = 200 μm. Immunofluorescence showing differentiated GSCs expressing GFAP or βIII tubulin (middle and below). Scale bar = 50 μm. d, e: The expression of *ISL2* in different patient-derived GSCs, as measured by western blotting (d) and qPCR (e). f, g: The expression of *ISL2* in different patient-derived GSCs and non-GSCs, as measured by western blotting (f) and qPCR (g). All data are expressed as the mean ± SD (three independent experiments). ^*^p < 0.05; ^**^p < 0.01; ^***^p < 0.001.

**Additional file 3: Supplementary Figure 3.** The expression of *ISL2*, cARF1, and *U2AF2* in GSCs after lentiviral-based transfection. a: The protein expression of *ISL2* after *ISL2* knockdown (left) or overexpression (right), as detected by western blotting. b: The mRNA expression of *ISL2* after *ISL2* knockdown (left) or overexpression (right), as detected by qPCR. c: The expression of cARF1 after cARF1 knockdown (left) or overexpression (right), as detected by qPCR. d: The mRNA expressions of *U2AF2* after *U2AF2* knockdown (left) or overexpression (right), as detected by qPCR. e: The protein expressions of *U2AF2* after *U2AF2* knockdown (left) or overexpression (right), as detected by western blotting. EV: empty vector, OE: overexpression, NC: negative control, KD: knockdown. All data are expressed as the mean ± SD (three independent experiments). ^***^p < 0.001.

**Additional file 4: Supplementary Figure 4.** *ISL2*-mediated GCM regulated the proliferation, invasion and angiogenesis of hBMECs. a: The MTS assays showed the inhibited cell viability of hBMECs after treatment with *ISL2* knockdown of GCM. b: The MTS assays showed the increased cell viability of hBMECs after treatment with *ISL2* overexpression of GCM. c, d: The EDU assay showed the proliferation of hBMECs after treatment with *ISL2* knockdown or overexpression GCM. Scale bar = 50 μm. e, f: A representative Transwell assay showed the invasion of hBMECs after treatment with *ISL2* knockdown or overexpression of GCM. Scale bar = 100 μm. j, h, i: A representative tube formation assay showing that the tubulogenesis of hBMECs after treatment with *ISL2* knockdown or overexpression of GCM. Scale bar = 100 μm. EV: empty vector, OE: overexpression, NC: negative control, KD: knockdown. All data are expressed as the mean ± SD (three independent experiments). ^**^p < 0.01; ^***^p < 0.001.

**Additional file 5: Supplementary Figure 5.** The correlation between the mRNA expression of *CD31* and *U2AF2* /circRNA ARF1/miR-342–3p/*ISL2* feedback loop in glioma specimens. a: Correlation between the mRNA expression of *CD31* and *ISL2*. b: Correlation between the mRNA expression of *CD31* and miR-381-3p. c: Correlation between the mRNA expression of *CD31* and cARF1. d: Correlation between the mRNA expression of *CD31* and *U2AF1*.

**Additional file 6: Supplementary Figure 6.** The direct function of candidate genes on the GSC properties. a. MTS assays showed overexpression of *ISL2*, cARF1 or *U2AF2* promote the cell viability of GSCs. b, c. The EDU assay showed the proliferation of GSCs after overexpression of *ISL2*, cARF1 or *U2AF2*. Scale bar = 50 μm. b, d: A representative Transwell assay showed the invasion of GSCs after overexpression of *ISL2*, cARF1 or *U2AF2*. Scale bar = 100 μm. All data are expressed as the mean ± SD (three independent experiments). **p < 0.01; ***p < 0.001.

**Additional file 7: Supplementary Table 1.** Relationship of ISL2 expression to clinical features of glioma patients.

**Additional file 8: Supplementary Table 2.** Clinical information of the primary glioma stem-like cells.

**Additional file 9: Supplementary Table 3.** siRNA sequences.

**Additional file 10: Supplementary Table 4.** PCR Primers.

**Additional file 11.**

## Data Availability

The datasets obtained and analyzed during the current study were made available from the corresponding authors through request.

## Abbreviations

circRNAs: Circular RNAs
GSCs: Glioma stem cells
ceRNAs: Competitive endogenous RNAs
RBP: RNA binding protein
hBMECs: Human brain microvessel endothelial cells
ECM: Endothelial cell medium
GCM: Glioma conditioned medium
IHC: Immunohistochemistry
ELISA: Enzyme-linked immunosorbent assay
ChIP: Chromatin immunoprecipitation
RIP: RNA immunoprecipitation
IDH: Isocitrate dehydrogenase
CGGA: Chinese Glioma Genome Atlas
TCGA: The Cancer Genome Atlas
LGG: Lower grade glioma
GBM: Glioblastoma
GSEA: Gene set enrichment analysis
MST: Median survival times
ARF1: ADP ribosylation factor 1
qRT-PCR: Real-Time Quantitative Reverse Transcription PCR
