# Supplementary material for: The U2AF2 /circRNA ARF1/miR-342–3p/ISL2 feedback loop regulates angiogenesis in glioma stem cells
Source: J Exp Clin Cancer Res. 2020 Sep 7;39:182. doi: 10.1186/s13046-020-01691-y (PMC7487667; doi:10.1186/s13046-020-01691-y)
Supplement: Supplementary file 7 — Additional file 7: Supplementary Table 1. Relationship of ISL2 expression to clinical features of glioma patients. [file 13046_2020_1691_MOESM7_ESM.docx]

**Supplementary Table 1.** Relationship of ISL2 expression to clinical features of glioma patients

| **Clinical features** | | **Samples**  **(*n* = 70)** | **ISL2 expression*** | | ***P* value** |
| --- | --- | --- | --- | --- | --- |
|  |  |  | **Low (*n* = 21)** | **High (*n* = 49)** |  |
| **Sex** | Male | 36 | 10 | 26 | P=0.1509 |
|  | Female | 34 | 11 | 23 |  |
| **Age** | ≤ 50 | 31 | 9 | 21 | P=0.9451 |
|  | > 50 | 39 | 12 | 27 |  |
| **IDH status** | Wild | 42 | 8 | 34 | **P=0.0143** |
|  | Mutant | 28 | 13 | 15 |  |
| **WHO grade** | II | 20 | 11 | 9 | **P=0.0028** |
|  | III | 25 | 8 | 17 |  |
|  | IV | 25 | 2 | 23 |  |

*: ISL2 expression was detected by immunohistochemistry and evaluated according to the German immunohistochemical score. High expression was defined as score ≥4.
