# Supplementary material for: The U2AF2 /circRNA ARF1/miR-342–3p/ISL2 feedback loop regulates angiogenesis in glioma stem cells
Source: J Exp Clin Cancer Res. 2020 Sep 7;39:182. doi: 10.1186/s13046-020-01691-y (PMC7487667; doi:10.1186/s13046-020-01691-y)
Supplement: Supplementary file 8 — Additional file 8: Supplementary Table 2. Clinical information of the primary glioma stem-like cells. [file 13046_2020_1691_MOESM8_ESM.docx]

Supplementary Table 2. Clinical information of the primary glioma stem-like cells

|  | GSC205 | GSC207 | GSC306 | GSC307 | GSC406 | GSC408 |
| --- | --- | --- | --- | --- | --- | --- |
| Gender | Male | Male | Female | Male | Male | Female |
| Age | 65 years old | 63 years old | 64 years old | 62 years old | 60 years old | 58 years old |
| Location | Right frontal lobe | Left insula | Left frontal lobe | Right parietal lobe | Right occipital lobe | Right temporal lobe |
| Pathological diagnosis | Astrocytoma | Astrocytoma | Anaplastic astrocytoma | Anaplastic astrocytoma; | Glioblastoma | Glioblastoma |
| WHO grade | II | II | III | III | IV | IV |
| Ki-67 | 35% (+) | 40% (+) | 55% (+) | 50% (+) | 60% (+) | 60% (+) |
