# Supplementary material for: The U2AF2 /circRNA ARF1/miR-342–3p/ISL2 feedback loop regulates angiogenesis in glioma stem cells
Source: J Exp Clin Cancer Res. 2020 Sep 7;39:182. doi: 10.1186/s13046-020-01691-y (PMC7487667; doi:10.1186/s13046-020-01691-y)
Supplement: Supplementary file 9 — Additional file 9: Supplementary Table 3. siRNA sequences. [file 13046_2020_1691_MOESM9_ESM.doc]

| **Primer** | **Forward (5’-3’)** | **Reverse (5’-3’)** |
| --- | --- | --- |
| ISL2-KD1 | UAACAAUAGAAUAAUUACGAA | CGUAAUUAUUCUAUUGUUAUU |
| ISL2-KD2 | AUAAAUAACAAUAACUUACGU | GUAAGUUAUUGUUAUUUAUUG |
| cARF1-KD1 | AUUUCAGCUUCAACGUGGAAA | UUUCCACGUUGAAGCUGAAAU |
| cARF1-KD2 | UAACUAUUUCAGCUUCAACGU | ACGUUGAAGCUGAAAUAGUUA |
| U2AF2-KD1 | AACAAAAUGUUUAAUGAGCAA | GCUCAUUAAACAUUUUGUUGU |
| U2AF2-KD2 | AUAUAAACACGCUAUGUUCCU | GAACAUAGCGUGUUUAUAUUU |
| siRNA-NC | UUCUUCGAAGGUGUCACGUTT | ACGUGACACCUUCGAAGAATT |

**Supplementary Table 3. siRNA sequences**
