# Supplementary material for: The U2AF2 /circRNA ARF1/miR-342–3p/ISL2 feedback loop regulates angiogenesis in glioma stem cells
Source: J Exp Clin Cancer Res. 2020 Sep 7;39:182. doi: 10.1186/s13046-020-01691-y (PMC7487667; doi:10.1186/s13046-020-01691-y)
Supplement: Supplementary file 10 — Additional file 10: Supplementary Table 4. PCR Primers. [file 13046_2020_1691_MOESM10_ESM.doc]

**Supplementary Table 4. PCR Primers**

| **Primer** | **Forward (5’-3’)** | **Reverse (5’-3’)** |
| --- | --- | --- |
| ISL2 | CTGCAAGCGGGACTACGTC | CACTCGATGTGGTACACGC |
| VEGFa | AGGGCAGAATCATCACGAAGT | AGGGTCTCGATTGGATGGCA |
| ARF1 | TGGTCAGGACAAGATTCG | CTCAGCAGCATTCATAGC |
| cARF1 | GTGTCGTGGAACCTCTTAC | GTGTTCTGGAAGTAGTGGC |
| U2AF2 | CGGCAGCTCAACGAGAATAAA | GGGAACGAATCAGTCCACCG |
| β-actin | CATGTACGTTGCTATCCAGGC | CTCCTTAATGTCACGCACGAT |

**qPCR Primers**

| **Primer** | **Forward (5’-3’)** | **Reverse (5’-3’)** |
| --- | --- | --- |
| VEGFa | CTAGGAAAATCGACCAGATGCC | AGCGCGTGTTGCAGGTCTTGGAT |
| U2AF2 | TATGTGCCTGGGGTTGTGTC | TGGCATTCTTGGCTCCCAC |

**ChIP qPCR Primers**
